# Discerning evolutionary trends in post-translational modification and the effect of intrinsic disorder: Analysis of methylation, acetylation and ubiquitination sites in human proteins

**DOI:** 10.1371/journal.pcbi.1006349

**Published:** 2018-08-10

**Authors:** Mohanalakshmi Narasumani, Paul M. Harrison

**Affiliations:** Department of Biology, McGill University, Montreal, QC, Canada; University of Texas at Austin, UNITED STATES

## Abstract

Intrinsically disordered regions (IDRs) of proteins play significant biological functional roles despite lacking a well-defined 3D structure. For example, IDRs provide efficient housing for large numbers of post-translational modification (PTM) sites in eukaryotic proteins. Here, we study the distribution of more than 15,000 experimentally determined human methylation, acetylation and ubiquitination sites (collectively termed ‘MAU’ sites) in ordered and disordered regions, and analyse their conservation across 380 eukaryotic species. Conservation signals for the maintenance and novel emergence of MAU sites are examined at 11 evolutionary levels from the whole eukaryotic domain down to the ape superfamily, in both ordered and disordered regions. We discover that MAU PTM is a major driver of conservation for arginines and lysines in both ordered and disordered regions, across the 11 levels, most significantly across the mammalian clade. Conservation of human methylatable arginines is very strongly favoured for ordered regions rather than for disordered, whereas methylatable lysines are conserved in either set of regions, and conservation of acetylatable and ubiquitinatable lysines is favoured in disordered over ordered. Notably, we find evidence for the emergence of new lysine MAU sites in disordered regions of proteins in deuterostomes and mammals, and in ordered regions after the dawn of eutherians. For histones specifically, MAU sites demonstrate an idiosyncratic significant conservation pattern that is evident since the last common ancestor of mammals. Similarly, folding-on-binding (FB) regions are highly enriched for MAU sites relative to either ordered or disordered regions, with ubiquitination sites in FBs being highly conserved at all evolutionary levels back as far as mammals. This investigation clearly demonstrates the complex patterns of PTM evolution across the human proteome and that it is necessary to consider conservation of sequence features at multiple evolutionary levels in order not to get an incomplete or misleading picture.

## Author summary

Eukaryotic proteins can be parsed into sections that are ‘ordered’ (having a fixed three-dimensional structure) or ‘disordered’. The latter do not take on a fixed structure during some of their functioning. Proteins are often chemically modified in cells after translation. Such modifications alter the functioning of the protein, occur at specific amino-acid residues, and there is a wide diversity of them. Disordered regions of proteins are efficient housing for these modifications. Here, we have studied three abundant and important types of such modification (methylation, acetylation, and ubiquitination). We have exploited a new large mass of protein sequences for eukaryotes to analyse the evolutionary appearance and conservation of amino-acid residues that are modified in this way in humans. We discover that there is enough information in this new data to discern ‘signals’ of conservation and emergence of these modifications over eukaryotic evolution.

## Introduction

Intrinsically disordered regions (IDRs) in proteins were initially discovered as long stretches of amino acids in proteins that remain unfolded under physiological conditions [1, 2]. IDRs can be functional despite this absence of a well-defined three-dimensional structure, and have caused a re-examination of the protein structure-function paradigm [1–4]. They are involved in numerous biological functions [2, 4–8] and their improper functioning leads to various disease conditions [7, 9–11]. Bioinformatical studies have shown that long (>30 residues) IDRs are common in eukaryotic proteins (33% of them on average) and occur much less in archaea (2% of proteins) and eubacteria (4%) [12–14]. In addition, Ward *et al*. reported that long IDRs (>30 residues) in yeast proteins are associated with transcription regulation and cell signalling [12]. The amino-acid sequences of IDRs contain compositional bias and low sequence complexity [15]. Many computational tools have been developed to annotate disordered regions in amino acid sequences [16–21], facilitating the distinction between ordered and disordered regions.

In many proteins, IDRs exhibit low amino-acid sequence conservation [22] and tandem repeats are more abundant in IDRs than in ordered regions [23, 24]. Insertions and deletions are more common in IDRs [25, 26] and they contain more amino acid substitutions than the ordered regions of the same proteins [22]. Furthermore, some disordered regions in proteins show conservation for chemical composition, but not detailed amino-acid sequence conservation [27]. Studies on the evolution of ordered and disordered regions have revealed that disordered regions generally evolve differently from ordered regions, but in some cases similarly to ordered regions [22, 26–31]. Hence, understanding the evolution of disordered regions in comparison to ordered regions has been challenging.

IDRs are involved in protein-protein interaction [11], including binding to kinases [32], transcription factors [33], and translation inhibitors [34], and they also mediate interaction with nucleic acids [33, 35]. Numerous receptors and enzymes with disordered regions acquire structure when binding to a partner molecule [4, 36–38]. Proteins with such folding on binding (FB) regions exhibit high specificity and low affinity towards a partner molecule [1, 39]. Compared to other disordered regions, they are enriched in hydrophobic residues, and positively charged amino acids [40] and are more conserved [31]. Post-translational modifications (PTMs) can induce their disorder-to-order transitions [41] [42]. Furthermore, PTMs in disordered regions have a significant role in signalling and regulation [42]. Experimental and computational studies suggest that PTMs including phosphorylation methylation and ubiquitination are enriched within IDRs, [6, 7, 42–45] whereas analysis of acetylation has shown contradictory results [46]. Furthermore, the phosphorylation sites present in disordered regions have been suggested to facilitate the evolution of transcriptional regulation [45, 47, 48]. Methylation, Acetylation, and Ubiquitination (abbreviated here collectively as ‘MAU’) are the three major PTMs, next to phosphorylation and glycosylation, which regulate the function of many eukaryotic proteins. Crosstalk between MAU sites facilitates complex regulatory programs in both histone and non-histone proteins [49]. However, the evolution of MAU sites in IDRs across eukaryotic species is not well understood [50–53]. Therefore, a comparative study on the conservation of human MAU site residues in ordered and disordered regions will illuminate their importance across the eukaryotic domain. Analysis of conservation across a large panel of genome-sequenced eukaryotes can give us more comprehensive insights into the evolutionary history of PTMs [45, 47, 48], while avoiding issues of data set completeness that may be a problem for experimental analysis of a variety of multi-cellular species.

We have performed a large-scale analysis of >15,000 experimentally-verified MAU sites from the ordered and disordered regions of >7,000 human proteins. We compiled four such data sets for both ordered and disordered regions: *(i)* methylated arginines, *(ii)* methylated lysines, *(iii)* acetylated lysines and *(iv)* ubiquitinated lysines. We studied the distribution and conservation of MAU-site residues in ordered and disordered regions across 380 eukaryotic organisms. Conservation signals for the maintenance and novel emergence of MAU sites were analysed at 11 evolutionary levels from the whole eukaryotic domain down to the level of the ape superfamily. We observed significant conservation attributable to lysine and arginine PTMs in both ordered and disordered regions across the 11 levels, and also some signals for the novel emergence of new MAU sites. Furthermore, we have pinpointed trends for biologically important subsets of IDRs, such as FB regions and prion-like domains. For example, we observed that MAU and other PTM sites are highly enriched in FB regions relative to both ordered and disordered regions generally and at evolutionary depths back as far as the emergence of the mammal class.

## Methods

### PTM datasets

Human proteins with experimentally-verified PTM sites were retrieved from dbPTM [54], PHOSIDA [55] and PhosphositePlus [56] databases as of November 2015. We focused on the evolutionary behaviour of Methylation, Acetylation and Ubiquitination sites (‘MAU sites’). Redundant annotations for PTMs were removed. This resulted in 1,009 lysine and 1,676 arginine methylation sites, 10,044 acetylation sites and 14,396 ubiquitination sites. We also comparatively analysed the distribution of serine, threonine and tyrosine phosphorylation sites, and other rarer PTMs (but not their evolutionary conservation).

### Eukaryotic proteomes

Complete proteomes of 380 eukaryotic organisms were downloaded from ENSEMBL [57], UniProt [58] and NCBI RefSeq [59] databases. The organisms were separated into eleven different taxonomic levels that provide a range of focus on the human: eukaryotes, metazoan, deuterostomes, chordates, vertebrates, mammals, tetrapods, eutherians, supraprimates, primates, and apes. Human proteins with experimentally-verified folding on binding regions (FB regions) were obtained from the IDEAL database [60].

### Sequence analysis

Phylogenetic trees of the eukaryotic organisms were drawn with Evolview [60] using Newick-format files generated by phyloT (https://phylot.biobyte.de/) [61]. Human orthologs in eukaryotic organisms were identified using the reciprocal best hit method with BLASTP and e-value threshold <1e-04 [62]. Multiple sequence alignment of human proteins with MAU sites and their orthologs in the 380 organisms was performed using ClustalOmega [63]. For the evolutionary analysis, human proteins with an orthologue in at least one of the organisms in a clade are included and the human proteins without an orthologue in at least one of the organisms are discarded. We used ZORRO, a probabilistic masking program to evaluate the alignment quality of individual positions [64]. In doing this, the aligned positions with low ZORRO score were discarded, and the positions within the recommended score range of five to ten were retained for conservation analysis. For comparison, the alignment program KMAD was also applied in some cases [65].

Enrichment analyses of gene ontology (GO) molecular function categories was performed using the GOrilla tool to identify GO terms enriched in different clades [66].

### Identification of ordered and disordered regions in proteins

We performed protein BLASTP [version 2.2.28] [62] against the ASTRAL non-redundant protein domain database (95% identity threshold) [67]. We used PDB atom records of proteins from ASTRAL domain database to identify the experimentally validated position of ordered regions in human proteins and the disordered regions in human proteins were annotated with DISOPRED and IUPRED per-residue prediction scores, using default parameters [18, 19]. Since ASTRAL domains are experimentally validated structures, we considered the region given by ASTRAL BLAST hits as ordered region for the cases that are also predicted as disordered. To keep the analysis and presentation of results manageable, regions un-classified in this way were not analysed.

Human prion-like proteins are annotated disordered regions that have a bias for asparagine or glutamine residues (using the fLPS program [68], run with default parameters except for a binomial P-value threshold of ≤1e-10, as used in previous studies [69–71]).

### Conservation & statistical analysis

A Python script was written to find the conserved MAU sites in ordered and disordered regions by calculating the completely conserved lysine/arginine residues in the multiple sequence alignment at each clade. Newly-emerged conserved residues are those that are completely conserved in a clade but not across a more ancient, wider clade. To test the significance of conservation, we performed enrichment analysis of the conserved MAU-site residues at each evolutionary level as subsets of the total sets of conserved residues of the same type, with appropriate corrections for multiple hypotheses.

Hypergeometric probability tests were used to find these enrichments of MAU-site residues in ordered and disordered regions for the different evolutionary levels. A Bonferroni correction for multiple hypothesis testing was applied for all tests for a given background population. The details of the enrichment calculations are given in the introductory page of S1 File. All enrichment and statistical analyses are performed using the R language [72].

## Results and discussion

First, we overview the distribution of methylation, acetylation and ubiquitination (MAU) sites in ordered and disordered regions, and include some specific analysis and discussion of MAU sites in folding-on-binding (FB) regions, prion-like proteins and homopeptides (which are common features of disordered regions [73]).

Then, we examine the effect of MAU sites on the evolutionary behaviour of lysine and arginine residues. To what extent do MAU sites drive the conservation of these residues and the appearance of new conserved residues at different points in eukaryotic evolution? Is there evidence for the appearance of new conserved lysines in evolutionarily old proteins because of MAU site status?

These questions are examined for each of methylation, acetylation and ubiquitination separately in turn. In doing so, we also consider the effects of: (i) allowing mutation to other possible residue types for the same modification (e.g., allowing mutation between arginine and lysine for methylation); (ii) alignment quality on the results (through applying the program ZORRO, as described in *Methods*); (iii) removal of histones (which are known to have high levels of MAU).

The evolution of MAU sites is also specifically examined for histones, and for folding-on-binding proteins as subsets. Finally, we briefly consider the evolutionary behaviour of sites that are *‘multiple-MAU’* (i.e., that can have more than one different type of MAU modification).

### Distribution of MAU sites in ordered and disordered regions

**Fig 1:**
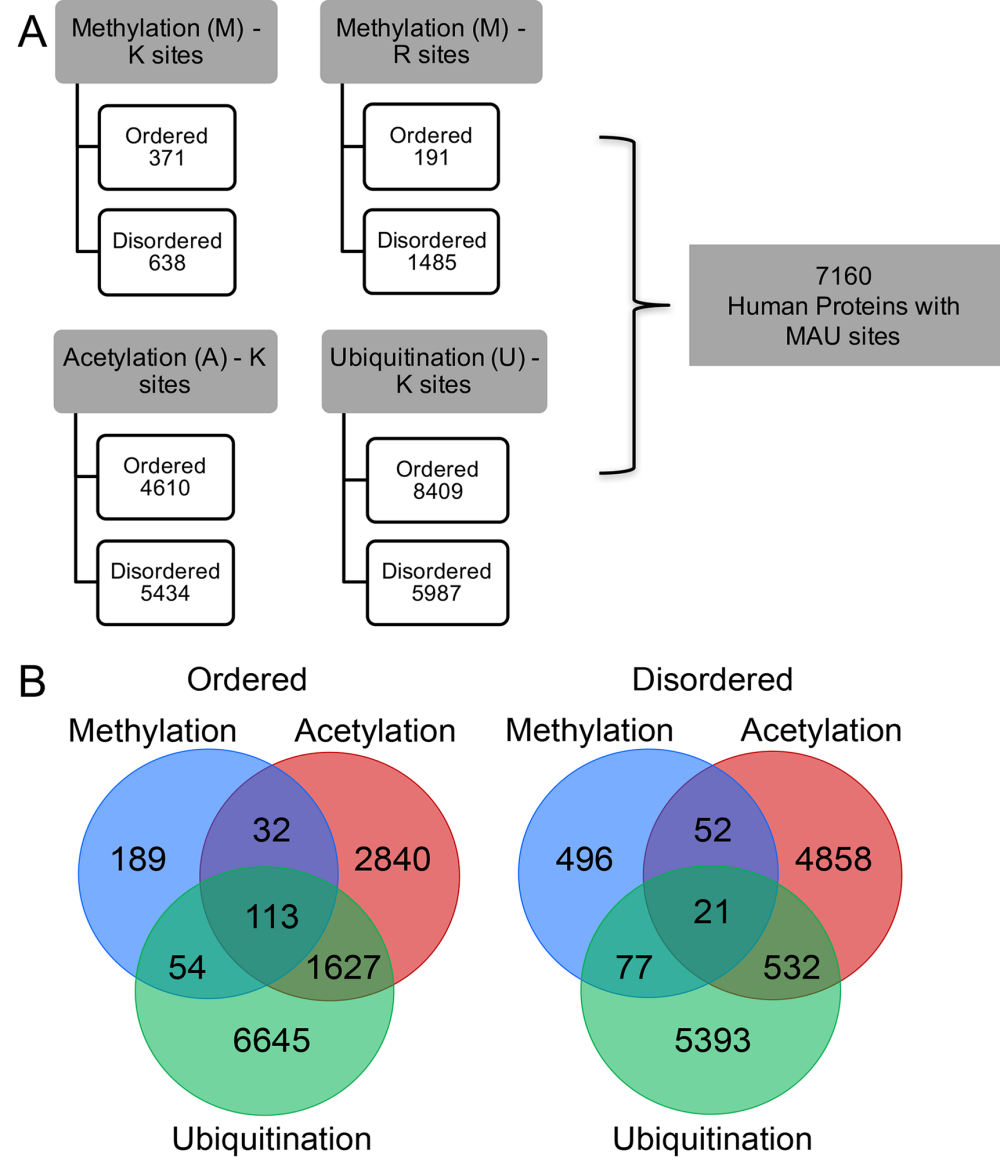
Overview of the number of methylation, acetylation and ubiquitination sites and the coincidence of different MAU types at the same residue in ordered and disordered regions. (A) total number of MAU sites in ordered and disordered regions of 7160 human proteins showing that the higher number of MAU sites in disordered regions than in ordered regions and ubiquitination sites show preference for ordered regions. (B) Venn diagram illustrates the co-incidence of MAU (i.e., how many can have two or three different MAU at the same residue) in ordered and disordered regions.

The MAU site contents in the ordered and disordered regions are summarized in Fig 1A. Specific lysine residues can be sites for multiple PTMs, including MAU (Fig 1B). For MAU sites in ordered and disordered regions, the observed overlap between acetylation and ubiquitination sites correlates with an established regulatory relationship [74], and it is also interesting to note the high proportion of methylation sites (~51%) specifically in ordered regions that have other PTMs, in comparison to any other MAU in either ordered or disordered regions (Fig 1B).

In general, PTM sites have been reported to be abundant in the disordered regions of eukaryotic proteins [7, 75]. However, not all PTMs show a preference for disordered regions. We examined the distribution in ordered and disordered regions of human proteins of experimentally-verified MAU sites, along with phosphorylation sites for comparison (as listed in *Methods*).

**Fig 2:**
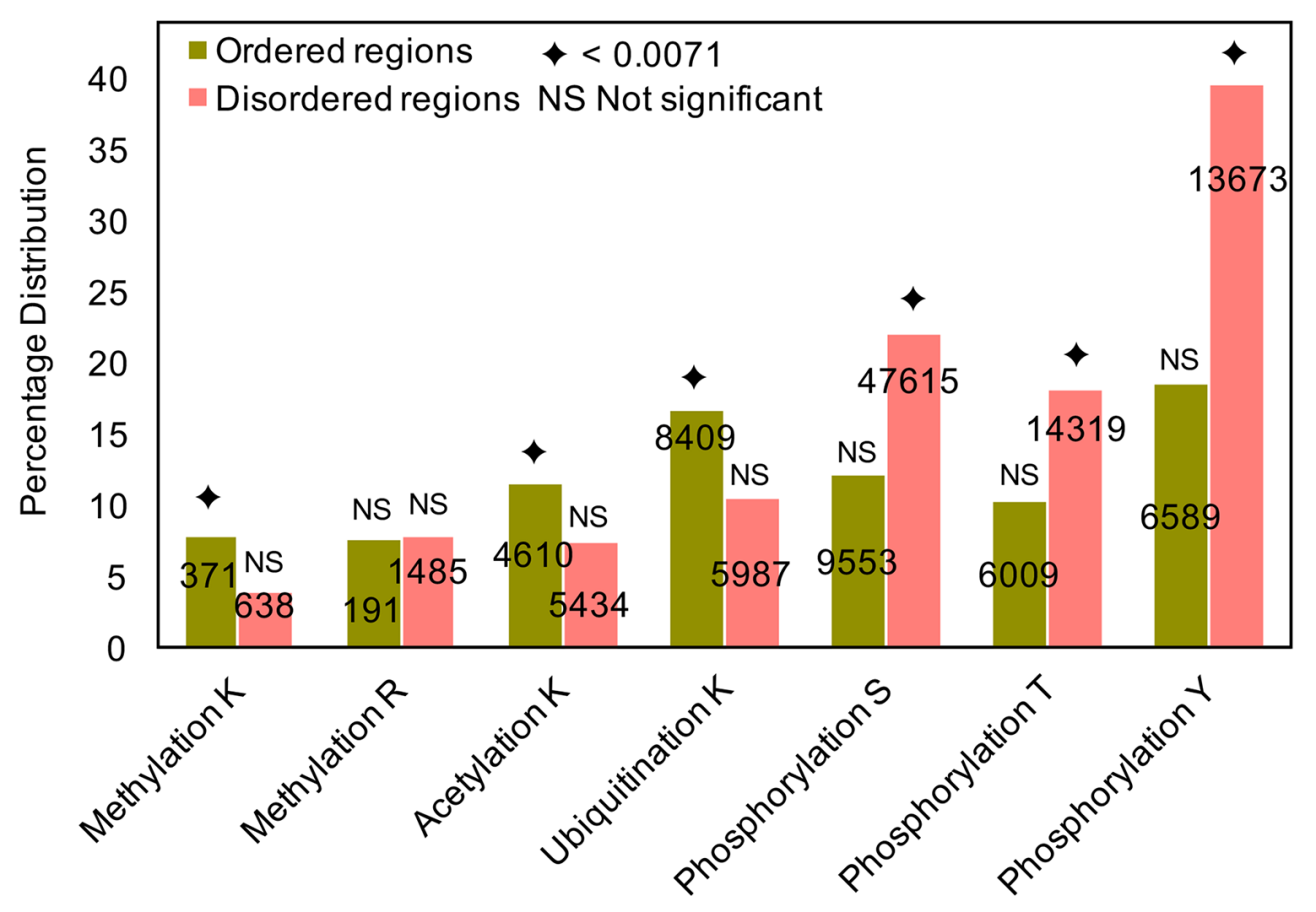
Percentage distribution of MAU and phosphorylation sites in ordered and disordered regions of human proteins. Percentages of MAU and phosphorylation sites (out of the total number of residues of the same type) in ordered and disordered regions of the human proteins analysed. The total number of each site present in ordered (olive green) and disordered (peach) regions are given in the centre of the bar. The hypergeometric distribution is used to identify the enrichment of MAU-modified residues in (dis)ordered regions in all lysines/arginines present in both ordered and disordered regions, with the total set of MAU sites as background population, and the diamond symbol on top of the bar indicates the corrected P-value (0.0071) for significant enrichment of PTMs in ordered and disordered regions, and NS represents non-significant enrichment.

We observe that acetylation and ubiquitination sites and methylated lysine sites generally have a significant preference for ordered regions (Fig 2). It is known that lysine methylation in disordered regions blocks site-specific lysine ubiquitination to increase protein half-life [76]. This may contribute to the relative abundance of ubiquitination sites in ordered regions. In comparison, phosphorylation sites prefer disordered regions, as expected [7, 75] (Fig 2).

Previous studies have suggested that MAU sites are enriched in disordered regions [6, 7, 42–44] and acetylated lysines have no preference for either ordered or disordered regions [46]. In contrast, our analysis here shows that experimentally-verified MAU lysines are significantly relatively enriched in ordered regions rather than in disordered ones, whereas the opposite is true for phosphorylation sites (Fig 2).

### FB regions as display areas for PTMs

**Fig 3:**
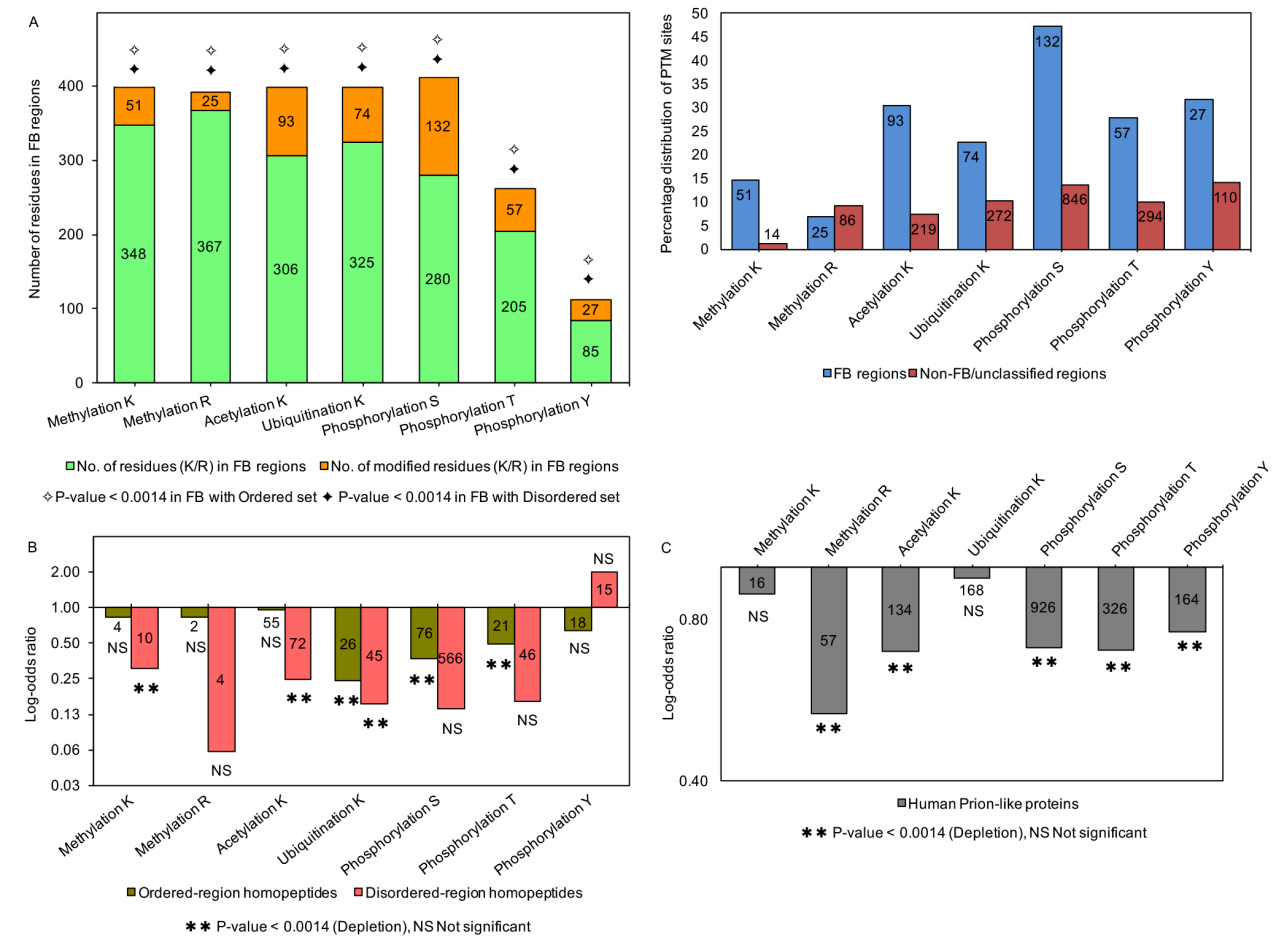
Distribution of PTM sites in ordered and disordered regions of human proteins for various subsets of the data. (A) Distribution of MAU and phosphorylation sites in folding on binding (FB) regions and the percentage distribution of sites in FB non-FB/unclassified regions. Enrichment analysis is performed for the FB set as a sample of total ordered or disordered regions. Due to the limited experimental data, other PTM sites were detected only at very low levels or were not present: nitrosylated cysteines 2 sites, O-linked glycosylation (serine, 1 site and threonine, 5 sites), prenylated cysteine (2 sites), sulfated tyrosine (2 sites) and sumoylated lysines (24 sites), whereas carboxylation, myristoylation, palmitoylation sites are not present in the FB regions. We used hypergeometric probability tests to perform the enrichment/depletion analyses of PTM sites in FB regions. The critical P-value to test the significance is P<0.0014 (to correct for multiple hypotheses). (B) Distribution of MAU and phosphorylation sites in homopeptides. The enrichment and depletion analyses are calculated for homopeptides present in the ordered (olive green) and disordered (peach) regions. The statistical test and critical P-value is as for part (A). (C) Distribution of MAU and phosphorylation sites in Human prion-like proteins (grey). Enrichment analysis is performed for lysines or arginines in the prion-like protein set as a sample of total lysines or arginines in the disordered set, as appropriate. The statistical test and critical P-value is as for part (B).

FB regions in proteins are known to interact with multiple and diverse partners [1, 39], and are associated with PTMs [41, 42]. Previously, we found that FB regions are more conserved than contiguous disordered regions that are not known to exhibit disorder-to-order transition [31]. We have analysed the enrichment of MAU sites and other PTMs in FB regions (in 172 human proteins, data taken from the IDEAL database [77]). Phosphorylation sites are highest in number in FB regions, followed by MAU sites (Fig 3A).

We observed that the major PTMs phosphorylation, methylation, acetylation, and ubiquitination are highly significantly enriched in FB regions treated as a sample either of ordered or of disordered regions (Fig 3A). In addition, two other less numerous PTMs namely O-linked glycosylation on threonines (P-values≤3E-05) and sumoylation on lysines (P-value≤6.7E-15) are significantly enriched in FB regions treated as a sample of either ordered or disordered regions (not depicted in the figure). Hence, MAU / phosphorylation site enrichment is a distinctive feature of FB regions relative to other (dis)ordered regions. Furthermore, we calculated the percentage distribution of MAU and phosphorylation sites in FB and non-FB/unclassified regions, and these sites show preference for FB regions, however the number of sites are higher in non-FB/unclassified regions (Fig 3A).

PTMs have been reported to induce disorder-to-order transition and facilitate binding to multiple partners [42]. In addition, PTM sites and ‘multiple-MAU’ sites (*i*.*e*., individual sites that can have multiple different MAU modifications) have been previously reported to show a preference for molecular recognition features (MoRFs) [44]. MoRFs are short (10–70 residues) structured regions within disordered regions, that are thought to undergo disorder-to-order transition on partner binding [78], whereas FB regions are of varying length within both ordered and disordered regions. We analysed the enrichment of multiple-MAU sites within FB regions (Table J in S1 File). We found a highly significant enrichment, treated as a sample of either ordered or disordered regions (P<1e-20). FB regions could be involved in many significant functions due to the prevalence of long disordered regions (>50 residues) in eukaryotic proteins [79]. Indeed, FB proteins with multiple-MAU sites such as flap endonuclease 1 (FEN1), α-synuclein, HMG-I and p53 are involved in DNA/RNA binding. For example, acetylation regulates the activity of FEN1 through p300 [80] and N-terminal acetylation leads to the α-helical oligomerization of α-synuclein [81]. Generally, FB regions are known to be involved in many interactions with high specificity and low affinity towards a partner molecule. Hence, FB PTMs could be crucial for facilitating these interactions.

### PTMs are depleted in homopeptides and prion-like proteins

Homopeptide repeats are common in eukaryotic proteins, and they tend to occur in disordered regions [82]. These repeats occur in a variety of nucleic-acid–binding domains linked to signalling and transcriptional processes [83]. We calculated the occurrence of PTMs in homopeptides (≥3 amino acids) in ordered and disordered regions. Among the major PTMs, a higher proportion of serine phosphorylation and lysine acetylation sites are present in the homopeptides of disordered regions (Fig 3B). However, enrichment/depletion analyses show that MAU sites are generally significantly depleted in both ordered- and disordered-region homopeptides, although phosphorylated tyrosines may be enriched in disordered-region homopeptides (Fig 3B). Other PTMs analysed do not show significant enrichment/depletion (i.e., P-values are non-significant); this might be due to their very limited experimental data. We suggest that the homopeptide lack of PTMs is due to the rapid evolution of amino-acid repeats [84], and also because they do not well accommodate required sequence motifs.

The intrinsically disordered nature of prion-like proteins and the role of PTMs such as N-glycosylation in changing the conformation and stability of prion proteins [42, 85–87] motivated us to study PTM occurrence in 1269 human prion-like proteins. We performed the analyses as mentioned above (Fig 3C). As for homopeptides, there is a general trend for significant depletion. We hypothesize that PTMs may get in the way of regular side-chain hydrogen-bonding patterns that are essential for prion amyloid formation. Notably also, prion-like proteins do not show a significantly high proportion of N-glycosylation sites, even though they tend to be N-rich (i.e., P-values are non-significant).

### Evolutionary behaviour of MAU sites at eleven evolutionary levels

The main goal of this work is to reveal to what extent the evolutionary behaviour of lysine and arginine amino acids is driven by MAU post-translational modification and by presence in intrinsic disorder. To this end, we analysed the evolutionary sequence variation of experimentally verified methylation (lysine: 1009 and arginine: 1676), lysine acetylation (10,044) and lysine ubiquitination (14,396) sites in human proteins.

**Fig 4:**
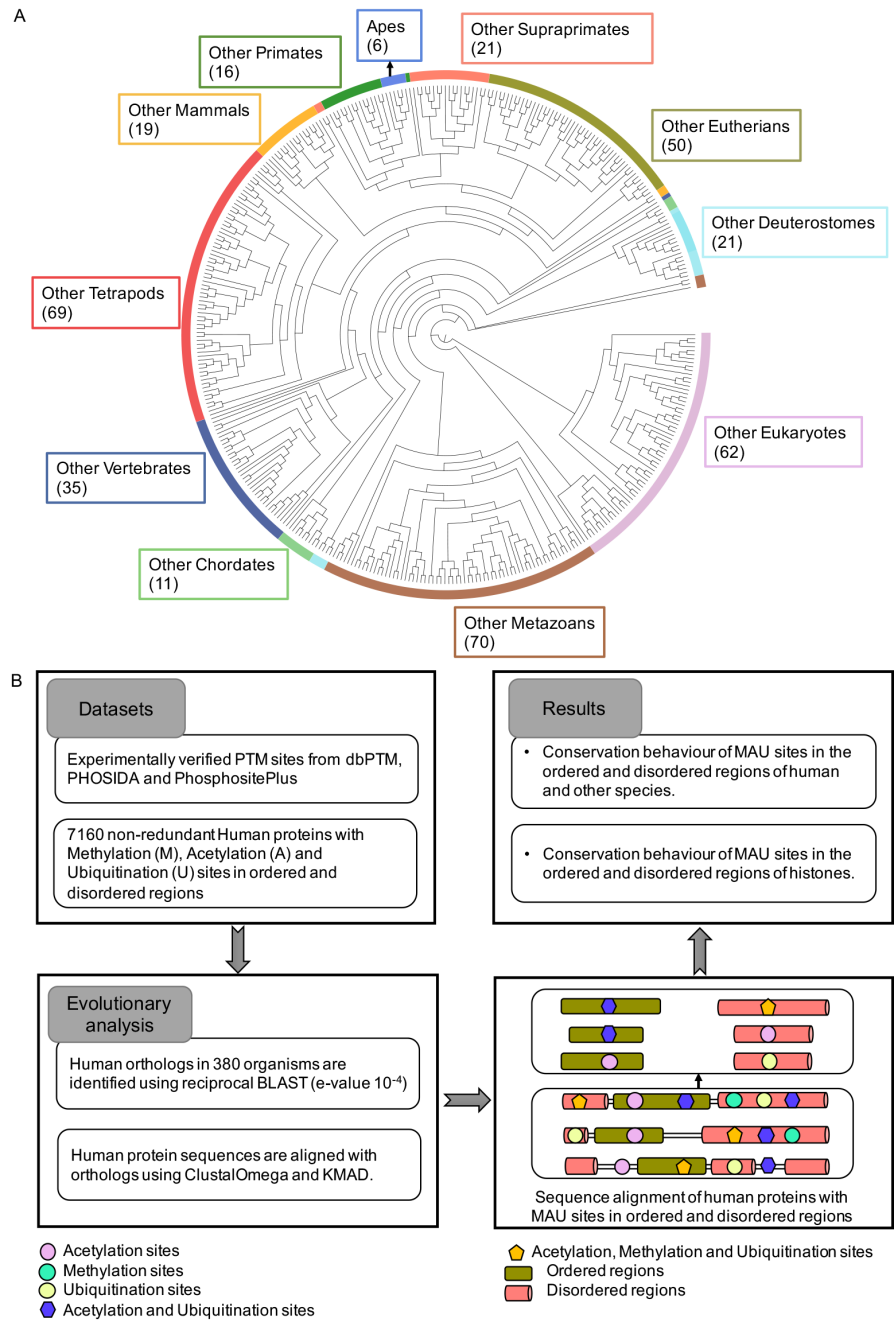
Organismal phylogeny and pipeline. (A) Organismal phylogenetic tree of eukaryotes separated into eleven clades and the total number of organisms for each is given in brackets. (B) Pipeline for the conservation analysis. MAU sites conserved in ordered and disordered regions are considered as two separate datasets.

**Fig 5:**
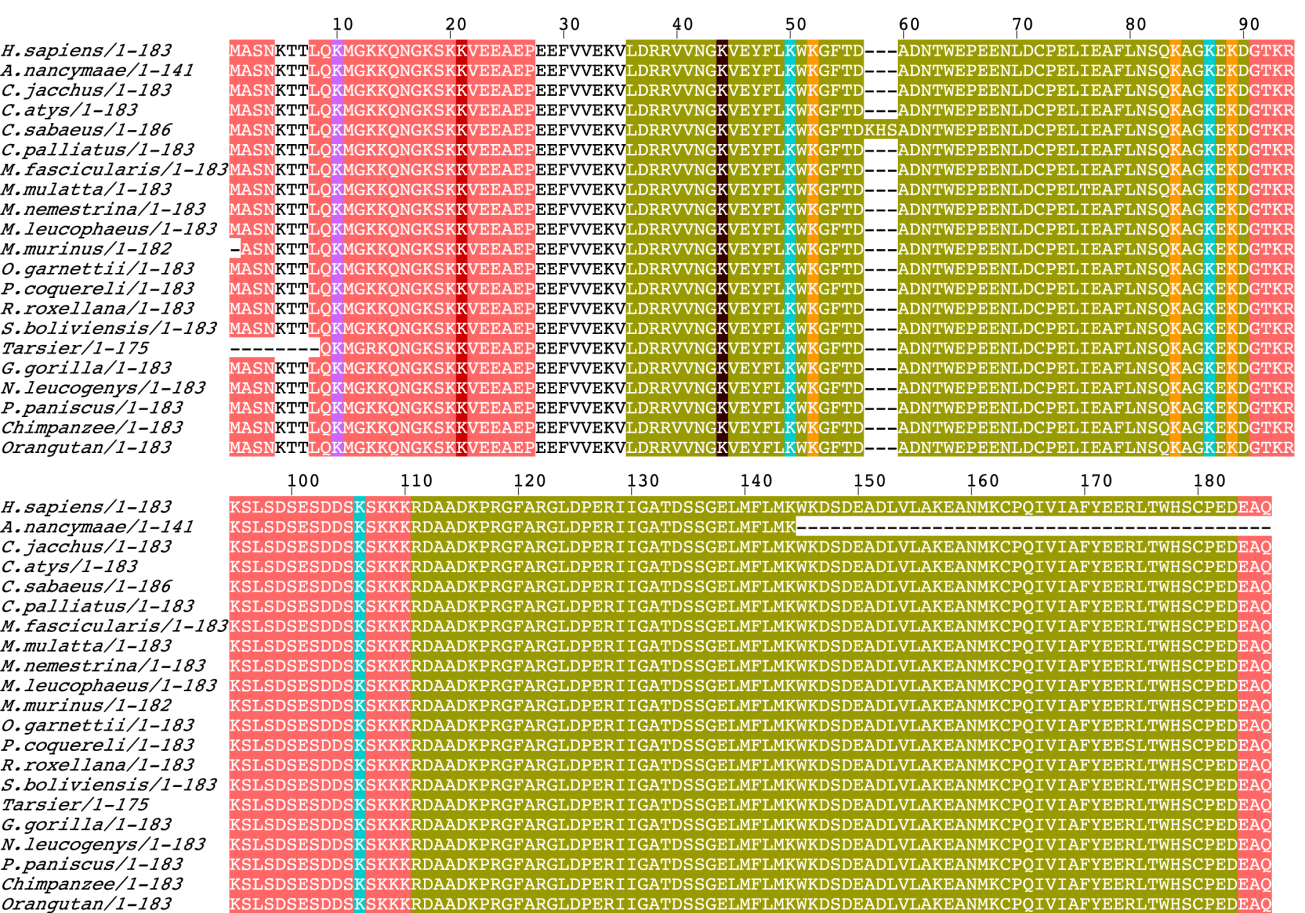
Example of a protein with methylation, acetylation and ubiquitination sites in ordered and disordered regions. Multiple sequence alignment of human chromobox protein homolog 3 and its primate orthologs, depicted using JalView [88], showing methylation, acetylation (purple) and ubiquitination (yellow) sites in ordered (green) and disordered (peach) regions. The sites with both acetylation and methylation sites are highlighted in brown, sites with both acetylation and ubiquitination sites are highlighted in cyan and the sites with acetylation, methylation and ubiquitination sites are highlighted in red.

We analysed the conservation trends at eleven evolutionary levels: (i) Apes, (ii) Primates, (iii) Supraprimates (primates + rodents + lagamorphs), (iv) Eutherians, (v) Mammals, (vi) Tetrapods, (vii) Vertebrates, (viii) Chordates, (ix) Deuterostomes, (x) Metazoans, and (xi) Eukaryotes (all 380 eukaryotes species examined) (Fig 4A). Conservation of MAU-site residues was investigated in the ordered and disordered regions across the 380 eukaryotic organisms using the pipeline of methods illustrated in Fig 4B. An illustrative example of a protein alignment (for ‘human chromobox protein homolog 3’) indicating the positions of MAU sites in ordered and disordered regions is shown in Fig 5. When we talk about conservation of PTM sites in the following analysis, it is the conservation for the amino-acid residues that is under consideration, and not for PTMs explicitly. It is discovered below that there is sufficient sequence information to discover conservation signals that indicate the maintenance and emergence of new MAU sites during the evolutionary ancestry of humans.

**Fig 6:**
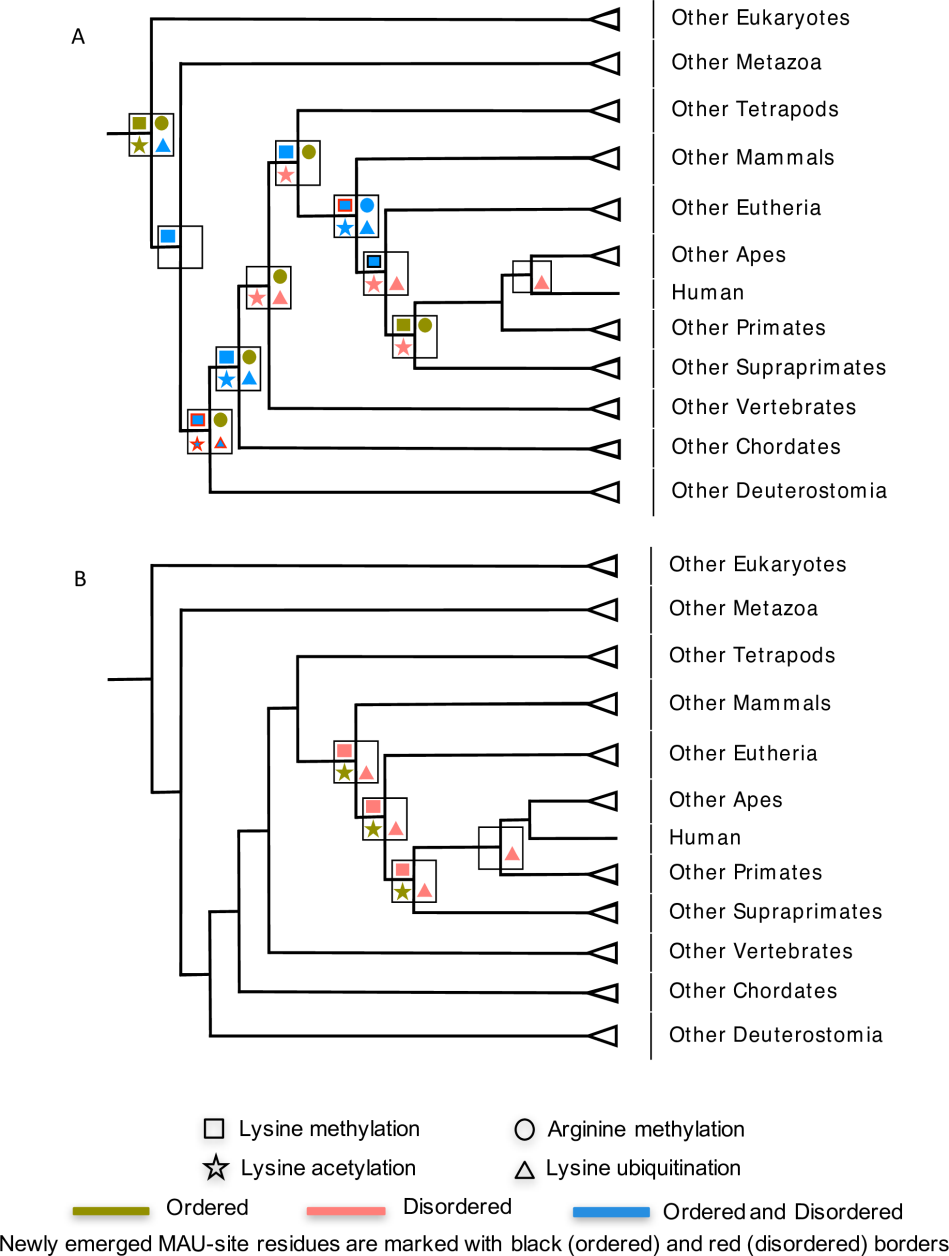
Summary of significantly enriched conserved MAU sites in ordered and disordered regions at 11 evolutionary clades. Evolutionary levels with significant enrichment (after correction for multiple hypotheses) are labelled with four different shapes: lysine methylation (square), arginine methylation (circle), lysine acetylation (star) and ubiquitination (triangle) sites. The ordered and disordered regions with enriched MAU sites are labelled in olive green and peach respectively and the sites with significant enrichment in both disordered and ordered regions are coloured blue. Where there are conservation signals for newly emerged MAU sites in ordered and disordered regions the symbols are marked with black and red borders respectively. The results are depicted in more detail (with P-values, specific thresholds and total numbers of sites) in S1 File. Table A in S1 File is for the total data set, and Table B in S1 File is for histones.

We examined the degree of conservation of arginines and lysines that are human MAU sites at each of the 11 evolutionary levels. We analysed: *(i)* the MAU site residues that are conserved (out of the total number of conserved arginines and lysines) for each of these 11 clades, and *(ii)* the MAU site residues that are newly emerged residues for that specific clade and are conserved right across it. To test the significance of conservation, we performed enrichment analysis of the conserved MAU sites at each evolutionary level, with appropriate corrections for multiple hypotheses. The fractions of conserved residues that are MAU sites at different evolutionary stages are shown on schematic species trees in S2 File. A summary schematic of the major results is shown in Fig 6.

In general, we found that, of proteins with MAU sites, 7.3% in ordered and 1.0% in disordered regions have conserved sites across all eukaryotes, with 3.0% of sites in ordered and 0.5% of sites in disordered being completely conserved in this way (Table 1). For example, the abundant eukaryotic DEAD-box protein p68 contains such completely conserved acetylatable (ordered: K-351) and ubiquitinatable (ordered: K-351, disordered: K-375) residues in both ordered and disordered regions. PTMs such as acetylation and ubiquitination are reported to regulate transcriptional coactivation and increase the stability of p68 [89]. The presence of conserved acetylation- and ubiquitination-site residues suggest an essential role of very specific PTMs in p68 across all eukaryotes.

**Table 1:** Percentages of human MAU-site residues in ordered and disordered regions that are conserved across all eukaryotes.

PTMs	Conserved residues		Proteins with conserved residues	
	Ordered	Disordered	Ordered	Disordered
All MAU sites	0.43%	0.02%	7.26%	1.01%
	(393/91535)	(32/155852)	(270/3719)	(48/4757)
Lysine Methylatable sites	5.06%	1%	4.78%	0.48%
	(18/356)	(4/593)	(11/230)	(2/417)
Arginine Methylatable sites	4.09%	0.16%	3.33%	0%
	(7/171)	(2/1289)	(5/150)	(0/637)
Acetylatable sites	2.59%	0.18%	5.03%	0.54%
	(113/4370)	(9/5011)	(97/1930)	(13/2387)
Ubiquitinatable sites	1.42%	0.43%	7.51%	1.21%
	(114/8029)	(24/5583)	(212/2823)	(35/2890)

#### Evidence for methylation as a driver of lysine conservation during eukaryotic evolution, and for the emergence of new lysine methylation sites

The fraction of conserved methylation-site lysine residues in each clade is shown in Figure A in S2 File, ordered regions being shown in green and, disordered regions in peach colour. The bubble size indicates the fraction of conservation. We find substantial evidence for significant conservation of lysine methylation sites across most of the 11 levels (P-values = 0.004 to 5e-21) except in apes, primates and vertebrates for ordered regions and apes, primates, supraprimates, vertebrates and across all eukaryotic organisms for disordered regions) (Figure A in S2 File, top and bottom left panels, and Table A in S1 File). This strong persistent conservation signal across most of the levels suggests that methylation is a major driver of lysine conservation in both ordered and disordered regions across eukaryote evolution.

In addition, for each clade we studied newly emerged lysines that are methylated in humans. By doing so, we can ask: Is lysine methylation also a driver for conservation for newly emerged lysine residues? We observed evidence for a significant enrichment of new lysine methylation sites in the ordered regions of eutherians (P = 6.9e-06), and in the disordered regions of mammals (P = 9.6e-04) and deuterostomes (P = 0.0011) (Figure A in S2 File / Table A in S1 File). Specifically, we observed a conservation signal for a significant number of evolutionarily new methylation sites appearing at various epochs in *old* proteins, *i*.*e*. proteins that emerged earlier in eukaryotic evolution. The significant enrichment of new sites in old proteins is similar to the above general results except that new sites are more highly enriched in the disordered regions of deuterostomes (P = 5e-04) (Table E in S1 File). Examples of such proteins in mammals are microtubule-associated protein tau and chromodomain Y-like protein (CDYL1). In tau proteins, methylatable residues K-163 and K-267 in disordered regions are conserved across mammals. K-267 residue methylation is reported to increase frequency of phosphorylation at S-262, and K-163 is identified as a site for both methylation and acetylation [90]. Moreover, methylation at these sites may play important roles in pathological conditions [90]. In mammals, in the protein CDYL1 methylatable K-135 in a disordered region is conserved, and is reported to regulate chromodomain binding to H3K9me3 [91]. These conservation signals for emergence of new lysine methylation sites suggest that clade-specific changes in modifying enzymes might cause progressive addition of more PTM sites to specific proteins in complex organisms.

All conservation signals for new emergent lysine methylation sites appear to be due to new sites in evolutionarily old proteins, *i*.*e*., there appears to be no significant contribution from new proteins (such as those arising from new gene duplications). This is also observed generally for all the MAU sites analysed further below.

We also examined the conservation of human lysine methylation sites while allowing for mutation to arginine (*i*.*e*., since arginines can also be methylated) and *vice versa*. This analysis also yields significant conservation signals at various evolutionary levels, with a few differences (Table B in S1 File). For example, specifically in eutherians, a signal for the emergence of new sites is observed in both ordered and disordered regions (Table B in S1 File). This indicates that methylated lysine sites could have been mutated to arginines in the epoch after eutherian emergence. Furthermore, in general the conservation analyses of aligned positions for human lysine methylation sites after applying the alignment quality filtering program ZORRO give similar results, but with increased significance (Table C in S1 File). Also, overall, there is little difference in the results upon removal of histones (Table D in S1 File), with just three results switching significance status in three of the analysed levels. In addition, we checked the effect of using an alternative alignment tool called KMAD, that has some features designed to apply to alignment of disordered proteins [65] (Table K in S1 File). This tool produced considerably less aligned positions overall at all evolutionary levels, but led to increased significance or acquisition of significance in the enrichments detected for 9 of the 11 levels, and decreases in significance for two of them (Deuterostomes and Metazoan). We also calculated the significant conservation of methylation sites in the disordered regions predicted by IUPRED software (Table L in S1 File), for comparison. IUPRED annotates fewer disordered regions than DISOPRED, however only one significance result changes (conservation at the primate level becomes significant) (Table K in S1 File).

#### Arginine methylation conservation is highly favoured in ordered regions across human evolutionary descent in eukaryotes

Arginine methylation has been extensively studied in both histones and non-histones, and generally involved in signal transduction, mRNA splicing, transcription factors and DNA repair (reviewed in [92]). Protein arginine methyltransferases have been identified in many non-mammalian organisms such as invertebrate chordates, arthropods and nematodes [92]. We find here evidence that right across eukaryotic evolution human methylated arginine sites have had significant conservation, almost exclusively in ordered regions (Figure B in S2 File, top left panel and Table A in S1 File). The human methylated arginines in ordered regions show a higher fraction of conservation than in disordered regions at almost all evolutionary levels. There are no significant conservation signals for the emergence of new methylated arginine sites during eukaryotic evolution. However, methylated arginine residues, when allowed to mutate to lysine, show potential emergence of new sites in metazoans, indicating potential allowance of such mutation (Table B in S1 File). Similar conservation results are obtained for IUPRED-predicted disordered regions, with additional enrichment in metazoans (Table K in S1 File). In addition, filtering for alignment quality using ZORRO or application of the KMAD tool yields similar results as for methylated Ks, *i*.*e*., increased and more pervasive significance, with additional enrichments in clades such as primates, eutherians, tetrapods and vertebrates. In general, since such quality filtering gives higher scoring for conserved positions, ordered regions tend to gain higher scores than disordered regions; however, generally in our analyses we see further significant conservation in disordered regions as well (Table C in S1 File). Also, similar results are obtained here when histones are removed from the data sets (Table D in S1 File).

In the analysis for newly emerged arginine methylation sites at various evolutionary levels, we looked specifically for a conservation signal indicating the emergence of new arginine methylation sites in evolutionarily old proteins (Table E in S1 File). We found a significant enrichment of such methylated arginines in the ordered regions of old proteins in tetrapods (P = 0.028). In tetrapods, these sites are identified in the ordered regions of proteins such as heterogeneous ribonucleoproteins hnRNP A2/B1 and A0. Arginine methylation sites in hnRNPs A2/B1 and hnRNP A0 are involved in cellular signaling and maturation of hnRNPs [93]. Furthermore, methylation-site arginine residues show conservation in the disordered regions of hnRNP H3 in tetrapods. hnRNP isoforms confer various splicing functions, and hnRNP is reported to transactivate tyrosine hydrolase gene transcription in tetrapods [94]. Thus, methylation-site arginine residue conservation correlates with their vital role in tetrapod hnRNPs.

#### Human acetylated lysines are favoured for significant conservation in disordered regions rather than in ordered regions across eukaryote evolution

To explore the conservation of lysine acetylation in ordered and disordered regions, we performed the same analysis as for methylation. Here, we find that human lysine acetylation sites are significantly enriched (P<0.00417) among conserved lysines in disordered regions at 7 out of the 11 evolutionary levels, more so than in ordered regions (4/11 levels) (Fig 6A). Notably, human acetylated lysines are significantly enriched among conserved lysines in disordered regions at several levels (P<1e-20) (Table A in S1 File and Figure C in S2 File, bottom left panel). Strong conservation evidence for the emergence of new disordered-region lysine acetylation sites is observed in Deuterostomes (P = 3e-21). There is no conservation signal for the emergence of new lysine acetylation sites in ordered regions at any evolutionary level (Fig 6A and Table A in S1 File), except that when mutation to other possible acetylation sites is allowed, it is observed in eutherians (Table B in S1 File).

Since there is a conservation signal for new lysine acetylation sites in disordered regions across deuterostomes, we examined a few proteins that may have acquired new sites in this evolutionary epoch. For example, new conservation at MAU sites is found in the disordered regions of CREB-binding protein (CBP) and p300 HAT. Six acetylated K residues are conserved in CBP IDRs. CBP is hypothesized to increase the acetylation of H3 and H4 histones and NcoA3 [95]. In p300 HAT, we found eight conserved acetylatable K residues in IDRs in the p300 loop region. The autoacetylation of K residues within this region is proposed to regulate the p300 HAT domain [95].

We analysed for evidence of new lysine acetylation sites in ‘new’ proteins (*i*.*e*. proteins that arose in each clade) and in ‘old’ proteins (*i*.*e*., proteins that arose earlier in evolution). We find conservation signals for new lysine acetylation sites in old proteins (Table E in S1 File) in both ordered (P = 0.0046) and disordered (P = 1e-21) regions of old proteins in deuterostomes.

As above for methylation, we checked whether the results are affected by the application of several criteria. Firstly, we compared the results to the case where the conservation of K acetylation sites as other residue types is allowed (*i*.*e*., substitution of acetylated K by A, G, M, S, or T; these are amino acids which can also be acetylated). We observed that the two datasets exhibit little or no difference (Table B in S1 File). This result suggests that the overall trend for conservation of human acetylation sites is robust to substitution of acetyl lysine to other possible acetylatable residues. In addition, IUPRED-predicted disordered regions show similar significances but with decreased significance in supraprimates, eutherians and tetrapods, and additional enrichment in primates (Table K in S1 File). As above, applying the ZORRO alignment quality filter or the KMAD tool, or removal of histones give similar or more highly significant enrichments.

#### Ubiquitination-site residue conservation is favoured in disordered regions of eukaryotic proteins

We analysed ubiquitination sites as above. We find that 4 out of 11 eukaryotic levels show significant enrichment of conserved ubiquitination sites in both ordered and disordered regions, and furthermore in apes, eutherians and vertebrates, only disordered regions exhibit significant conservation (P<0.0025) of these sites (Table A in S1 File and Figure D in S2 File). In deuterostomes, we found a significant signal for new sites in disordered regions (P<0.00417). Moreover, when we focused on potential new sites in evolutionarily old proteins, we found similar enrichment for disordered regions, with all the potential additional sites found in deuterostomes present in such old proteins (P<1e-10) (Table E in S1 File).

For example, the human ubiquitinated K-56 residue in IDRs is newly conserved in RNA helicase p68 across deuterostomes. The poly-ubiquitination of overexpressed p68 is reported in colorectal neoplasms [96]. Moreover, mutation of sumoylation sites is reported to increase polyubiquitination, therefore resulting in p68 aggregation [96]. In addition, ubiquitinatable K-207 is newly conserved across deuterostomes in the disordered regions of MCM3, an essential DNA replication licensing factor. K-207 in MCM3 is reported to be ubiquitinated by KEAP1 and KEAP1-mediated MCM3 ubiquitination sites are stated to be on predicted exposed surfaces of the C-terminal domain in MCM3 [97]. Such conservation suggests that these ubiquitinatable sites in the disordered regions could have facilitated macromolecular interactions since the dawn of deuterostomes.

Previously, for a much smaller data set, it has been observed that ubiquitination sites are more conserved than unmodified lysines in both ordered and disordered regions in mammals, whereas these sites are not more significantly conserved than unmodified sites in yeast [50]. Here, we discover that such conservation has been maintained throughout various stages of human eukaryotic ancestry. Also, we find a conservation signal for the emergence of new ubiquitination sites during deuterostome evolution (Fig 6A, Table A in S1 File). Furthermore, similar conservation results are observed for the IUPRED-predicted disordered regions but with loss of significance for two clades (Table K in S1 File). As above, filtering with the ZORRO program or application of the KMAD program (Tables C and L in S1 File) in general accentuate the conservation results with additional enrichments in several further clades, and removing histones makes little or no difference (Table D in S1 File).

### Conservation signals for MAU sites in histones

Histone proteins are highly conserved in all eukaryotes, and their regulatory activity is intimately linked to MAU and phosphorylation. These modifications provide several functions to histones and can modify nucleosome shape and stability. For example, acetylation and phosphorylation alter the charge of histone proteins. Methylation is more complex, i.e. lysine can be mono-, di- or tri-methylated, and ubiquitination provides a much larger covalent modification [98]. Most histone modifications occur within the disordered N-terminal tails, where they are linked to regulation of chromatin structure and recruitment of enzymes to reposition nucleosomes [98]. Furthermore, ordered regions of histones are highly conserved and modifications in these regions are also observed. Extensive study of the cross-talk between PTMs in histone tails has given rise to the term “histone code”, wherein histone tails exhibit sites for multiple PTM types and function in transcriptional regulation [41, 99]. Hence, we wished to compare the conservation behaviour of MAU sites in the ordered and disordered regions of histones.

We examined the MAU sites in histones that are significantly enriched in each clade. The percentage of histones in the total proteins analysed is 0.69%, which almost triples (to 1.74%) for proteins with conserved MAU sites across all eukaryotes. We found a similar pattern of significant conservation signals across three evolutionary levels (mammals, eutherians and supraprimates), *i*.*e*., for lysine methylation sites and ubiquitination sites in disordered regions, and for acetylation sites in ordered regions, (P-values = 2e-03 to 2.8e-07) (Table F in S1 File and Fig 6B).

#### Methylation site lysine residues in the disordered regions of linker H1 and H3 variants are conserved as far back as mammals

Histones have a significant enrichment for conserved methylation-site residues in disordered regions in mammalian, eutherian and supraprimates clade alignments (Table F in S1 File). Hence, we examined some individual cases for further perspectives. In mammals, we found a notable number of conserved methylation site residues in the disordered regions of Histone H1 variants H1.0 (K-12, K-102 and K-108) and H1.3 (K-17, K-107 and K-169) and of Histone H3 variants H3.2 and H3.3 (7 conserved site residues each). The linker histone H1.1 binds between the nucleosomes and is part of higher-order chromatin structure. H1 variant PTMs might be involved in modulating DNA binding [100]. Lysine acetylation in the H1 N-terminal region reduces H1 affinity to chromatin, and also recruits TAF1 to activate transcription [101]. In addition, lysine methylation in the N-terminal region of Histone H3 has been linked with strong cognitive abilities [102]. Thus, conservation of methylatable lysine residues in the disordered regions of histone H1 and H3 variants might facilitate cell-specific transcription and exhibit vital roles in neurodegenerative diseases.

#### Ubiquitination sites in H2A and H3 variants in mammalian histones

In mammalian histones, conserved ubiquitination-site lysines are significantly enriched in disordered regions (Table F in S1 File). The highest number of conserved ubiquitinatable site residues are observed in Histone H2A variants such as Histone H2A.1 (positions 120, 126, 128 and 130) and Histone H2A type 2-B (positions 119, 120, 125, 128 and 130). This could be linked to monoubiquitination being common in H2A and H2B, and present in all cells of higher organisms [103]. PTMs in intrinsically disordered histone tail domains have diverse functional impacts. For example, during spermatogenesis, proteasome-mediated degradation of histones may facilitate chromatin condensation [103]. Also, ubiquitinated H2A is involved in gene silencing and suppresses transcription initiation by inhibiting methylation of H3 at K-4 [103]. Hence, the results suggest that modifications on the disordered regions of histone variants that altered nucleosome stability were consolidated in the epoch of evolution since the dawn of mammals.

### Sites with multiple MAU PTMs

Multiple PTMs can occur on the same residue in a protein. Histone proteins are the best-known example of this; they have such ‘multiple-MAU’ sites in their N-terminal tail regions. The association between multiple-MAU sites and signalling is also observed in other proteins, *e*.*g*., α-tubulin, RNA polymerase II, p300/CBP and Cdc25C phosphatases [44]. PTM cross-talking at these sites such as between phosphorylation/acetylation, phosphorylation/sumoylation, hydroxylation/O-linked-glycosylation, and acetylation/ubiquitination has been reported [74, 99]. As shown in Fig 1B, our analysis shows the pronounced co-occurrence of acetylation and ubiquitination that plays a major regulatory role [74]. Previously it was shown that multiple-MAU sites show a strong preference for disordered regions [44].

We checked whether having multiple MAU modifications at one site is linked to increased sequence conservation for the 11 evolutionary levels. This would also be a further strong indicator that the conservation signals we have observed are due to conservation of PTMs at various evolutionary depths. PTM sites in human proteins with more than one MAU modification were separated into ordered (1836 sites) and disordered regions (676 sites). We found evidence for significant conservation of multiple-MAU sites in disordered regions in apes (P = 0.009) and supraprimates (P = 1e-05), and in ordered regions in apes (P = 0.006), eutherians (P = 9e-26), chordates (P = 1.5e-43) and across all eukaryotes (P = 3.5e-04). Also, there are conservation signals that appear due to the emergence of new conserved sites, *e*.*g*., in chordate, eutherian and supraprimate clades for ordered regions, and very high significance is found in supraprimates (P = 1e-82) for disordered regions (Table H in S1 File). Many of the P-values for these are smaller than the P-value for any relevant individual PTM enrichment, indicating potential increased conservation due to their multiple-MAU status.

### Ubiquitination is a major driver of conservation of lysines in folding-on-binding (FB) regions

Analysis of PTM sites in folding-on-binding regions showed that phosphorylation and MAU sites are significantly enriched (Fig 3A). So, we analysed the conservation of MAU sites in FB regions across the 11 evolutionary levels (Table I in S1 File). Here, we treated the FB regions as a sample of both ordered regions and disordered regions.

In eutherians, we observe significant enrichments of conserved lysines/arginines for all types of MAU in the FB regions (as samples of either disordered or ordered regions) (Table I in S1 File). Ubiquitination-site residues are the most enriched, with a persistence of enrichment back as far the mammalian clade (P = 4e-3), followed by acetylated lysines (Table I in S1 File). Examples of human proteins with ubiquitination-site residues in FB regions that have such conservation in mammals are: Myc proto-oncogene, histone H3.3, Ras-related C3 botulinum toxin substrate-1 (Rac1), and Protein CASC3.

For example, the Myc proto-oncogene, a transcription factor, is shown to undergo phosphorylation on T-58 and S-62 prior to its degradation by ubiquitination. The interaction of Fbw7 on T-58 is reported to promote degradation of Myc protein, and the mutation on this site results in decreased degradation [104]. The ubiquitinated K-18 in the FB region of the histone H3 tail is identified to mediate DNA methylation by interacting with the N-terminal regulatory domain of DNMT1 [105]. Furthermore, it has been reported that the ubiquitinated Rac1 might be involved in the internalization of Rac1 from peripheral membrane and relocation of Rac1 towards endocytic vesicles. In addition, mutations at evolutionarily conserved ubiquitination sites are identified to be enriched in cancer [105]. Therefore, these results suggest that the enriched ubiquitination sites in FB or disordered regions could be due to the shorter half-life of these proteins.

### Functional trends in MAU-site containing proteins

We checked for any interesting functional trends in the conservation data through examining Gene Ontology (GO) annotation [106]. Specifically, we were interested in the functional trends for proteins that have newly emerged conserved MAU-site residues at each evolutionary level. We see high enrichments for very general GO categories involved in ‘binding’, such as ‘nucleotide binding’, ‘RNA binding’, ‘protein binding’, ‘anion binding’, etc. These functional trends tend to be detectable from the tetrapod clade down to primates, but not so much outside of this range. These results tally well with a general role for MAU PTMs in modifying binding specificities and modalities (Figure A in S3 File). Interestingly, when compared to the GO category enrichments for the whole set of MAU-modified proteins (Figure B in S3 File), there are a few missing categories, *e*.*g*., ‘drug binding’, ‘organic cyclic compound binding’, and ‘microtubule binding’, suggesting that MAU sites on proteins with these functions do not undergo concerted changes in conservation across deep eukaryotic evolutionary time.

### Concluding remarks

**Fig 7:**
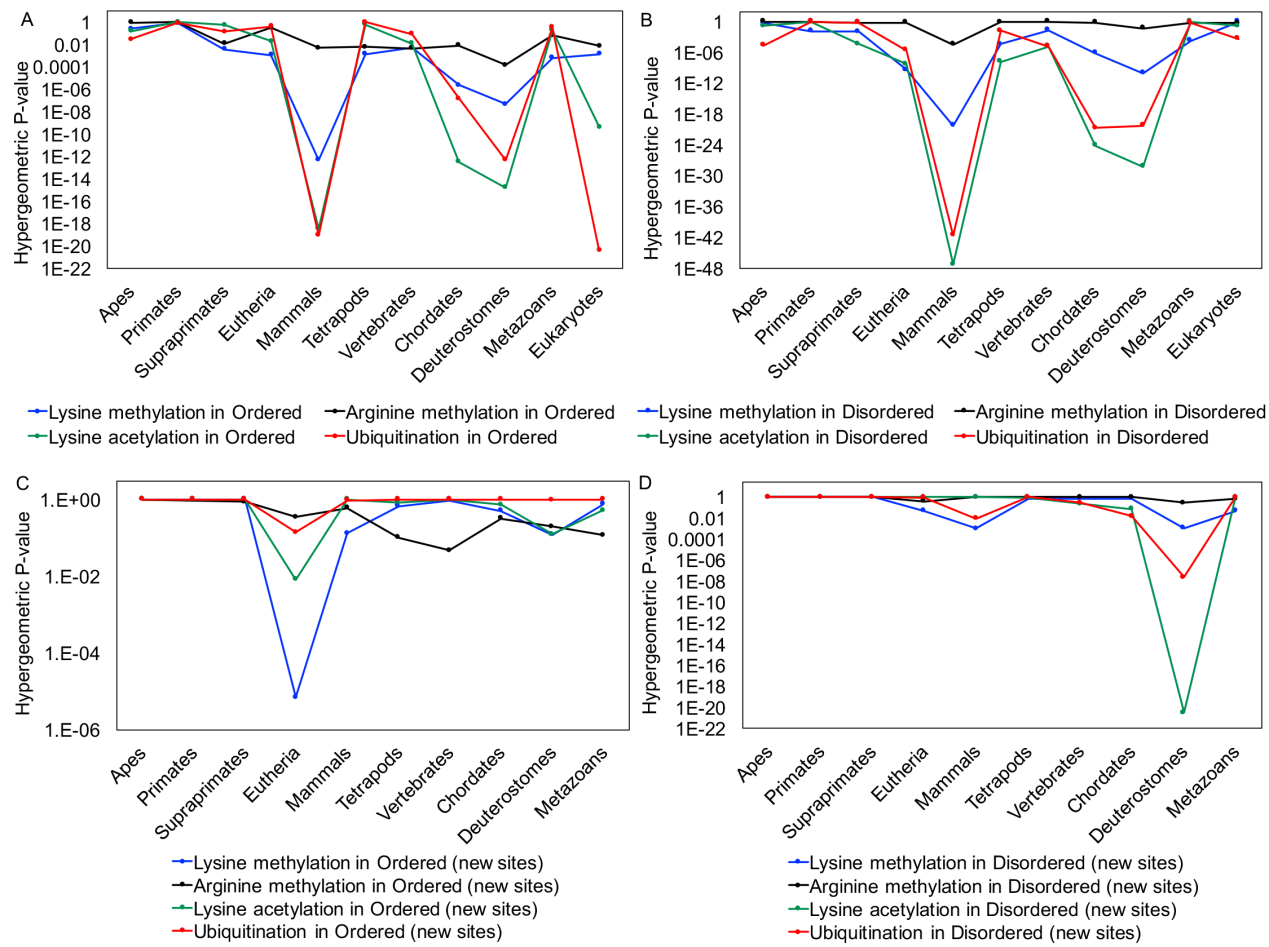
*The trends in enrichments for conserved PTM-site lysines and arginines that are maintained at each level (parts A and B) and newly-emerged (‘new sites’*, *parts C and D)*. The eleven evolutionary levels examined are on the horizontal axis. The P-values for enrichment are on the logarithmic vertical axis. Panels (A) and (C) are for Ordered regions, and (B) and (D) for Disordered regions.

Intrinsically disordered regions can house large numbers of post-translational modifications, such as the MAU sites which are the focus of this study. By examining for conservation of these sites in ordered and disordered regions separately, we have discovered that MAU is an important driver of arginine/lysine conservation throughout different stages of eukaryotic evolution, and that there is evolutionary evidence for key moments in human ancestry where new MAU sites have arisen in existing proteins, particularly during the epochs of deuterostome and eutherian evolution. The conservation signals for emergence of new PTM sites suggest that clade-specific changes in modifying enzymes might cause the progressive addition of more PTM sites to specific proteins in complex organisms. There is a surprising variety of conservation patterns for MAU-site residues when comparing disordered and ordered regions to each other. The four types of MAU site (methylatable K and R, acetylatable K and ubiquinatable K) each have distinct conservation patterns, with conservation of methylatable Rs being strongly favoured in ordered regions. In contrast, methylatable Ks are conserved in either set of regions, and conservation of acetylatable and ubiquitinatable Ks is favoured in disordered regions over ordered. The strongest conservation signals occur across the mammalian clade, indicating its appropriate use as a baseline conservation level for further analyses (Fig 7A and 7B), but for newly-emerged cases, the signals are strongest in other clades, indicating specific epochs of evolutionary emergence (Fig 7C and 7D). Distinct patterns of MAU-site evolution are observed in histones during eukaryote evolution, as compared to non-histones. However, removal of histones from the data makes little or no difference to the overall results. Also, in general filtering for alignment quality increases significances, in both ordered and disordered regions.

Examining the scenario where mutation to other possible MAU sites yields some interesting variant results. For example, a conservation signal for an emergent allowance of mutation between methylated arginine (R) and lysine (K) is observed in a certain epoch. This suggests that some sites switched between R and K and left a trace of this in the conservation pattern.

Folding-on-binding regions have highly significant enrichments in MAU sites (particularly ubiquitination sites) relative to other ordered or disordered regions, that persist back as far as mammalian emergence. Also, in some cases ‘multiple-MAU’ sites, i.e., sites that can be modified in any of the three ways, demonstrate highly significant conservation that is much more significant than for any corresponding single PTM type.

The number of conserved sites gets smaller when conservation is analysed for larger, wider clades. Conversely, however, the residues that are PTMed in human are a larger fraction of these deeply conserved residues, so significant conservation is still detected. This investigation demonstrates that analysis of conservation across a large panel of genome-sequenced eukaryotes can give us more comprehensive insights into the evolutionary history of PTMs, while avoiding issues of data set completeness that may be a problem for experimental analysis of such a variety of multi-cellular species. Also it is clear that we need to consider conservation of sequence features at multiple levels in order not to get an incomplete or misleading picture.

## Supporting information

S1 File File of supplementary tables detailing the enrichments of MAU sites in various sets. The contents of each table are described in a table listing on the first sheet of the file.
(XLSX)

S2 File Phylogenetic trees showing methylation, acetylation and ubiquitination sites conservation as other MAU site residue types in ordered and disordered regions at eleven evolutionary levels. The numbers in the bubbles are the fraction of conserved MAU sites with the number of conserved sites and P-value showing the enrichment of conserved sites in ordered and disordered regions at each level and the fraction of new conserved MAU sites with the number of new conserved sites in ordered and disordered regions at each level. **(A)** Fraction of conserved and new conserved lysine methylation sites and the number of conserved and new conserved lysine methylation sites in ordered and disordered regions. **(B)** Fraction of conserved and new conserved arginine methylation sites and the number of conserved and new arginine methylation sites in ordered and disordered regions. **(C)** Fraction of conserved and new conserved lysine acetylation sites and the number of conserved and new lysine acetylation sites in ordered and disordered regions. **(D)** Fraction of conserved and new conserved lysine ubiquitination sites and the number of conserved and new lysine ubiquitination sites in ordered and disordered regions.
(DOCX)

S3 File Gene Ontology enrichments. Summary of Gene Ontology (GO) category enrichments at different evolutionary levels. (A) Gene ontology category enrichments for proteins with newly emerged conserved MAU-site residues at each evolutionary level are depicted in heat map form. (B) Gene Ontology category for the whole set of MAU-modified proteins.
(DOCX)

## References

[1] Wright PE, Dyson HJ. Intrinsically unstructured proteins- re-assessing the protein structure-function paradigm. J Mol Biol. 1999;293:321–31. 10.1006/jmbi.1999.3110 10550212

[2] Dunker AK, Lawson JD, Celeste JB, Romero P, Oh JS, Oldfield CJ, et al Intrinsically disordered proteins. Journal of Molecular Graphics. 2001;19(1):26–59. 10.1016/s1093-3263(00)00138-8 11381529

[3] Tompa P. Intrinsically unstructured proteins. Trends in biochemical sciences. 2002;27(10):527–33. . 12368089 10.1016/s0968-0004(02)02169-2

[4] Dunker AK, Brown CJ, Lawson JD, Lakoucheva LM, Obradović Z. Intrinsic disorder and protein function. Biochemistry. 2002;41(21):6573–82. 12022860 10.1021/bi012159+

[5] Dunker AK, Silman I, Uversky VN, Sussman JL. Function and structure of inherently disordered proteins. Current opinion in structural biology. 2008;18(6):756–64. 10.1016/j.sbi.2008.10.002 . 18952168

[6] Xie H, Vucetic S, Iakoucheva LM, Oldfield CJ, Dunker AK, Uversky VN, et al Functional anthology of intrinsic disorder. 1. Biological processes and functions of proteins with long disordered regions. Journal of proteome research. 2007;6(5):1882–98. 10.1021/pr060392u ; PubMed Central PMCID: PMC2543138. 17391014 PMC2543138

[7] Xie H, Vucetic S, Iakoucheva LM, Oldfield CJ, Dunker AK, Obradovic Z, et al Functional anthology of intrinsic disorder. 3. Ligands, post-translational modifications, and diseases associated with intrinsically disordered proteins. Journal of proteome research. 2007;6(5):1917–32. 10.1021/pr060394e ; PubMed Central PMCID: PMC2588348. 17391016 PMC2588348

[8] Vucetic S, Xie H, Iakoucheva LM, Oldfield CJ, Dunker AK, Obradovic Z, et al Functional anthology of intrinsic disorder. 2. Cellular components, domains, technical terms, developmental processes, and coding sequence diversities correlated with long disordered regions. Journal of proteome research. 2007;6(5):1899–916. 10.1021/pr060393m ; PubMed Central PMCID: PMCPMC2588346. 17391015 PMC2588346

[9] Uversky VN, Oldfield CJ, Dunker AK. Intrinsically disordered proteins in human diseases: introducing the D2 concept. Annual review of biophysics. 2008;37:215–46. 10.1146/annurev.biophys.37.032807.125924 . 18573080

[10] Uversky VN, Oldfield CJ, Midic U, Xie H, Xue B, Vucetic S, et al Unfoldomics of human diseases: linking protein intrinsic disorder with diseases. BMC genomics. 2009;10 Suppl 1:S7 10.1186/1471-2164-10-S1-S7 ; PubMed Central PMCID: PMC2709268. 19594884 PMC2709268

[11] Weinreb PH, Zhen W, Poon AW, Conway KA, Lansbury PT Jr. NACP, a protein implicated in Alzheimer's disease and learning, is natively unfolded. Biochemistry. 1996;35(43):13709–15. 10.1021/bi961799n . 8901511

[12] Ward JJ, Sodhi JS, McGuffin LJ, Buxton BF, Jones DT. Prediction and functional analysis of native disorder in proteins from the three kingdoms of life. Journal of molecular biology. 2004;337(3):635–45. 10.1016/j.jmb.2004.02.002 . 15019783

[13] Dunker AK, Obradovic Z, Romero P, Garner EC, Brown CJ. Intrinsic protein disorder in complete genomes. Genome informatics Workshop on Genome Informatics. 2000;11:161–71. . 11700597

[14] Peng Z, Yan J, Fan X, Mizianty MJ, Xue B, Wang K, et al Exceptionally abundant exceptions: comprehensive characterization of intrinsic disorder in all domains of life. Cellular and molecular life sciences: CMLS. 2015;72(1):137–51. 10.1007/s00018-014-1661-9 . 24939692 PMC11113594

[15] Pedro Romero ZO, 1¥ Li Xiaohong,1‡ Garner Ethan C.,2† Brown Celeste J.,2 and Dunker A. Keith. Sequence Complexity of Disordered Protein. PROTEINS: Structure, Function, and Genetics. 2001;42. 10.1002/1097-0134(20010101)42:1<38::aid-prot50>3.0.co;2-3 11093259

[16] Prilusky J, Felder CE, Zeev-Ben-Mordehai T, Rydberg EH, Man O, Beckmann JS, et al FoldIndex: a simple tool to predict whether a given protein sequence is intrinsically unfolded. Bioinformatics. 2005;21(16):3435–8. 10.1093/bioinformatics/bti537 . 15955783

[17] Linding R, Russell RB, Neduva V, Gibson TJ. GlobPlot: Exploring protein sequences for globularity and disorder. Nucleic acids research. 2003;31(13):3701–8. ; PubMed Central PMCID: PMCPMC169197. 12824398 10.1093/nar/gkg519 PMC169197

[18] Dosztanyi Z, Csizmok V, Tompa P, Simon I. IUPred: web server for the prediction of intrinsically unstructured regions of proteins based on estimated energy content. Bioinformatics. 2005;21(16):3433–4. 10.1093/bioinformatics/bti541 WOS:000231360600018. 15955779

[19] Ward JJ, McGuffin LJ, Bryson K, Buxton BF, Jones DT. The DISOPRED server for the prediction of protein disorder. Bioinformatics. 2004;20(13):2138–9. 10.1093/bioinformatics/bth195 . 15044227

[20] Xue B, Dunbrack RL, Williams RW, Dunker AK, Uversky VN. PONDR-FIT: a meta-predictor of intrinsically disordered amino acids. Biochimica et biophysica acta. 2010;1804(4):996–1010. 10.1016/j.bbapap.2010.01.011 ; PubMed Central PMCID: PMCPMC2882806. 20100603 PMC2882806

[21] Garner E, Cannon P, Romero P, Obradovic Z, Dunker AK. Predicting Disordered Regions from Amino Acid Sequence: Common Themes Despite Differing Structural Characterization. Genome informatics Workshop on Genome Informatics. 1998;9:201–13. . 11072336

[22] Brown CJ, Takayama S, Campen AM, Vise P, Marshall TW, Oldfield CJ, et al Evolutionary rate heterogeneity in proteins with long disordered regions. Journal of molecular evolution. 2002;55(1):104–10. 10.1007/s00239-001-2309-6 . 12165847

[23] Szalkowski AM, Anisimova M. Markov models of amino acid substitution to study proteins with intrinsically disordered regions. PloS one. 2011;6(5):e20488 10.1371/journal.pone.0020488 ; PubMed Central PMCID: PMC3103576. 21647374 PMC3103576

[24] Jorda J, Xue B, Uversky VN, Kajava AV. Protein tandem repeats—the more perfect, the less structured. FEBS J. 2010;277(12):2673–82. 10.1111/j.1742-464X.2010.07684.x ; PubMed Central PMCID: PMCPMC2928880. 20553501 PMC2928880

[25] Light S, Sagit R, Sachenkova O, Ekman D, Elofsson A. Protein expansion is primarily due to indels in intrinsically disordered regions. Molecular biology and evolution. 2013;30(12):2645–53. 10.1093/molbev/mst157 . 24037790

[26] Brown CJ, Johnson AK, Daughdrill GW. Comparing models of evolution for ordered and disordered proteins. Molecular biology and evolution. 2010;27(3):609–21. 10.1093/molbev/msp277 ; PubMed Central PMCID: PMCPMC2822292. 19923193 PMC2822292

[27] Uversky VN. A decade and a half of protein intrinsic disorder: biology still waits for physics. Protein Sci. 2013;22(6):693–724. 10.1002/pro.2261 ; PubMed Central PMCID: PMCPMC3690711. 23553817 PMC3690711

[28] Tompa P. Intrinsically unstructured proteins evolve by repeat expansion. BioEssays: news and reviews in molecular, cellular and developmental biology. 2003;25(9):847–55. 10.1002/bies.10324 . 12938174

[29] Chen JW, Romero P, Uversky VN, Dunker AK. Conservation of intrinsic disorder in protein domains and families: I. A database of conserved predicted disordered regions. Journal of proteome research. 2006;5(4):879–87. 10.1021/pr060048x ; PubMed Central PMCID: PMC2543136. 16602695 PMC2543136

[30] Brown CJ, Johnson AK, Dunker AK, Daughdrill GW. Evolution and disorder. Current opinion in structural biology. 2011;21(3):441–6. 10.1016/j.sbi.2011.02.005 ; PubMed Central PMCID: PMCPMC3112239. 21482101 PMC3112239

[31] Narasumani M, Harrison PM. Bioinformatical parsing of folding-on-binding proteins reveals their compositional and evolutionary sequence design. Scientific reports. 2015;5:18586 10.1038/srep18586 ; PubMed Central PMCID: PMCPMC4683461. 26678310 PMC4683461

[32] Adkins JN, Lumb KJ. Intrinsic structural disorder and sequence features of the cell cycle inhibitor p57Kip2. Proteins. 2002;46(1):1–7. . 11746698 10.1002/prot.10018

[33] Chang JF, Phillips K, Lundback T, Gstaiger M, Ladbury JE, Luisi B. Oct-1 POU and octamer DNA co-operate to recognise the Bob-1 transcription co-activator via induced folding. Journal of molecular biology. 1999;288(5):941–52. 10.1006/jmbi.1999.2711 . 10329190

[34] Johansson J, Gudmundsson GH, Rottenberg ME, Berndt KD, Agerberth B. Conformation-dependent antibacterial activity of the naturally occurring human peptide LL-37. The Journal of biological chemistry. 1998;273(6):3718–24. . 9452503 10.1074/jbc.273.6.3718

[35] Tucker PA, Tsernoglou D, Tucker AD, Coenjaerts FE, Leenders H, van der Vliet PC. Crystal structure of the adenovirus DNA binding protein reveals a hook-on model for cooperative DNA binding. The EMBO journal. 1994;13(13):2994–3002. ; PubMed Central PMCID: PMCPMC395187. 8039495 10.1002/j.1460-2075.1994.tb06598.x PMC395187

[36] Cheng EH, Kirsch DG, Clem RJ, Ravi R, Kastan MB, Bedi A, et al Conversion of Bcl-2 to a Bax-like death effector by caspases. Science. 1997;278(5345):1966–8. . 9395403 10.1126/science.278.5345.1966

[37] Bidwell LM, McManus ME, Gaedigk A, Kakuta Y, Negishi M, Pedersen L, et al Crystal structure of human catecholamine sulfotransferase. Journal of molecular biology. 1999;293(3):521–30. 10.1006/jmbi.1999.3153 . 10543947

[38] Huang Y, Komoto J, Konishi K, Takata Y, Ogawa H, Gomi T, et al Mechanisms for auto-inhibition and forced product release in glycine N-methyltransferase: crystal structures of wild-type, mutant R175K and S-adenosylhomocysteine-bound R175K enzymes. Journal of molecular biology. 2000;298(1):149–62. 10.1006/jmbi.2000.3637 . 10756111

[39] Dunker AK, Garner E, Guilliot S, Romero P, Albrecht K, Hart J, et al Protein disorder and the evolution of molecular recognition: theory, predictions and observations. Pacific Symposium on Biocomputing Pacific Symposium on Biocomputing. 1998:473–84. . 9697205

[40] Meszaros B, Tompa P, Simon I, Dosztanyi Z. Molecular principles of the interactions of disordered proteins. Journal of molecular biology. 2007;372(2):549–61. 10.1016/j.jmb.2007.07.004 WOS:000249372200022. 17681540

[41] Dyson HJ, Wright PE. Intrinsically unstructured proteins and their functions. Nat Rev Mol Cell Bio. 2005;6(3):197–208. 10.1038/nrm1589 WOS:000227303200013. 15738986

[42] van der Lee R, Buljan M, Lang B, Weatheritt RJ, Daughdrill GW, Dunker AK, et al Classification of intrinsically disordered regions and proteins. Chemical reviews. 2014;114(13):6589–631. 10.1021/cr400525m ; PubMed Central PMCID: PMC4095912. 24773235 PMC4095912

[43] Pang CN, Hayen A, Wilkins MR. Surface accessibility of protein post-translational modifications. Journal of proteome research. 2007;6(5):1833–45. 10.1021/pr060674u . 17428077

[44] Pejaver V, Hsu WL, Xin FX, Dunker AK, Uversky VN, Radivojac P. The structural and functional signatures of proteins that undergo multiple events of post-translational modification. Protein Science. 2014;23(8):1077–93. 10.1002/pro.2494 WOS:000339664800007. 24888500 PMC4116656

[45] Holt LJ, Tuch BB, Villen J, Johnson AD, Gygi SP, Morgan DO. Global Analysis of Cdk1 Substrate Phosphorylation Sites Provides Insights into Evolution. Science. 2009;325(5948):1682–6. 10.1126/science.1172867 WOS:000270131800044. 19779198 PMC2813701

[46] Gao J, Xu D. Correlation between posttranslational modification and intrinsic disorder in protein. Pacific Symposium on Biocomputing Pacific Symposium on Biocomputing. 2012:94–103. ; PubMed Central PMCID: PMCPMC5120255. 22174266 PMC5120255

[47] Studer RA, Rodriguez-Mias RA, Haas KM, Hsu JI, Vieitez C, Sole C, et al Evolution of protein phosphorylation across 18 fungal species. Science. 2016;354(6309):229–32. 10.1126/science.aaf2144 WOS:000387816500045. 27738172

[48] Pearlman SM, Serber Z, Ferrell JE. A Mechanism for the Evolution of Phosphorylation Sites. Cell. 2011;147(4):934–46. 10.1016/j.cell.2011.08.052 WOS:000296902300024. 22078888 PMC3220604

[49] Yang XJ, Seto E. Lysine acetylation: codified crosstalk with other posttranslational modifications. Mol Cell. 2008;31(4):449–61. 10.1016/j.molcel.2008.07.002 ; PubMed Central PMCID: PMCPMC2551738. 18722172 PMC2551738

[50] Hagai T, Toth-Petroczy A, Azia A, Levy Y. The origins and evolution of ubiquitination sites. Mol Biosyst. 2012;8(7):1865–77. 10.1039/c2mb25052g . 22588506

[51] Lu L, Li Y, Liu Z, Liang F, Guo F, Yang S, et al Functional constraints on adaptive evolution of protein ubiquitination sites. Sci Rep. 2017;7:39949 10.1038/srep39949 ; PubMed Central PMCID: PMCPMC5215434. 28054638 PMC5215434

[52] Simonti CN, Pollard KS, Schroder S, He D, Bruneau BG, Ott M, et al Evolution of lysine acetylation in the RNA polymerase II C-terminal domain. Bmc Evol Biol. 2015;15 ARTN 35 10.1186/s12862-015-0327-z WOS:000350953800001. PMC4362643 25887984

[53] Drazic A, Myklebust LM, Ree R, Arnesen T. The world of protein acetylation. Bba-Proteins Proteom. 2016;1864(10):1372–401. 10.1016/j.bbapap.2016.06.007 WOS:000382272900010. 27296530

[54] Lee TY, Huang HD, Hung JH, Huang HY, Yang YS, Wang TH. dbPTM: an information repository of protein post-translational modification. Nucleic acids research. 2006;34(Database issue):D622–7. 10.1093/nar/gkj083 ; PubMed Central PMCID: PMCPMC1347446. 16381945 PMC1347446

[55] Gnad F, Gunawardena J, Mann M. PHOSIDA 2011: the posttranslational modification database. Nucleic acids research. 2011;39(Database issue):D253–60. 10.1093/nar/gkq1159 ; PubMed Central PMCID: PMCPMC3013726. 21081558 PMC3013726

[56] Hornbeck PV, Kornhauser JM, Tkachev S, Zhang B, Skrzypek E, Murray B, et al PhosphoSitePlus: a comprehensive resource for investigating the structure and function of experimentally determined post-translational modifications in man and mouse. Nucleic Acids Res. 2012;40(Database issue):D261–70. Epub 2011/12/03. 10.1093/nar/gkr1122 ; PubMed Central PMCID: PMCPMC3245126. 22135298 PMC3245126

[57] Yates A, Akanni W, Amode MR, Barrell D, Billis K, Carvalho-Silva D, et al Ensembl 2016. Nucleic Acids Res. 2016;44(D1):D710–6. Epub 2015/12/22. 10.1093/nar/gkv1157 ; PubMed Central PMCID: PMCPMC4702834. 26687719 PMC4702834

[58] Breuza L, Poux S, Estreicher A, Famiglietti ML, Magrane M, Tognolli M, et al The UniProtKB guide to the human proteome. Database (Oxford). 2016;2016. Epub 2016/02/21. 10.1093/database/bav120 ; PubMed Central PMCID: PMCPMC4761109. 26896845 PMC4761109

[59] O'Leary NA, Wright MW, Brister JR, Ciufo S, Haddad D, McVeigh R, et al Reference sequence (RefSeq) database at NCBI: current status, taxonomic expansion, and functional annotation. Nucleic Acids Res. 2016;44(D1):D733–45. Epub 2015/11/11. 10.1093/nar/gkv1189 ; PubMed Central PMCID: PMCPMC4702849. 26553804 PMC4702849

[60] He ZL, Zhang HK, Gao SH, Lercher MJ, Chen WH, Hu SN. Evolview v2: an online visualization and management tool for customized and annotated phylogenetic trees. Nucleic Acids Research. 2016;44(W1):W236–W41. 10.1093/nar/gkw370 WOS:000379786800039. 27131786 PMC4987921

[61] Letunic I, Bork P. Interactive tree of life (iTOL) v3: an online tool for the display and annotation of phylogenetic and other trees. Nucleic Acids Res. 2016;44(W1):W242–5. Epub 2016/04/21. 10.1093/nar/gkw290 ; PubMed Central PMCID: PMCPMC4987883. 27095192 PMC4987883

[62] Altschul SF, Gish W, Miller W, Myers EW, Lipman DJ. Basic local alignment search tool. J Mol Biol. 1990;215(3):403–10. Epub 1990/10/05. 10.1016/S0022-2836(05)80360-2 . 2231712

[63] Sievers F, Higgins DG. Clustal Omega, accurate alignment of very large numbers of sequences. Methods Mol Biol. 2014;1079:105–16. Epub 2013/10/31. 10.1007/978-1-62703-646-7_6 . 24170397

[64] Wu M, Chatterji S, Eisen JA. Accounting for alignment uncertainty in phylogenomics. PLoS One. 2012;7(1):e30288 Epub 2012/01/25. 10.1371/journal.pone.0030288 ; PubMed Central PMCID: PMCPMC3260272. 22272325 PMC3260272

[65] Lange J, Wyrwicz LS, Vriend G. KMAD: knowledge-based multiple sequence alignment for intrinsically disordered proteins. Bioinformatics. 2016;32(6):932–6. 10.1093/bioinformatics/btv663 WOS:000372975000019. 26568635 PMC4803389

[66] Eden E, Navon R, Steinfeld I, Lipson D, Yakhini Z. GOrilla: a tool for discovery and visualization of enriched GO terms in ranked gene lists. BMC bioinformatics. 2009;10:48 10.1186/1471-2105-10-48 ; PubMed Central PMCID: PMCPMC2644678. 19192299 PMC2644678

[67] Chandonia JM, Hon G, Walker NS, Lo Conte L, Koehl P, Levitt M, et al The ASTRAL Compendium in 2004. Nucleic Acids Res. 2004;32(Database issue):D189–92. Epub 2003/12/19. 10.1093/nar/gkh034 ; PubMed Central PMCID: PMCPMC308768. 14681391 PMC308768

[68] Harrison PM. fLPS: Fast discovery of compositional biases for the protein universe. Bmc Bioinformatics. 2017;18 Artn 476 10.1186/S12859-017-1906-3 WOS:000414990700001. 29132292 PMC5684748

[69] An L, Fitzpatrick D, Harrison PM. Emergence and evolution of yeast prion and prion-like proteins. Bmc Evolutionary Biology. 2016;16 ARTN 24 10.1186/s12862-016-0594-3 WOS:000369163700002. 26809710 PMC4727409

[70] An L, Harrison PM. The evolutionary scope and neurological disease linkage of yeast-prion-like proteins in humans. Biology direct. 2016;11 ARTN 32 10.1186/s13062-016-0134-5 WOS:000380191000001. PMC4960796 27457357

[71] Harbi D, Parthiban M, Gendoo DMA, Ehsani S, Kumar M, Schmitt-Ulms G, et al PrionHome: A Database of Prions and Other Sequences Relevant to Prion Phenomena. PloS one. 2012;7(2). ARTN e31785 10.1371/journal.pone.0031785 WOS:000302871500088. PMC3282748 22363733

[72] Team RC. R: A language and environment for statistical computing R Foundation for Statistical Computing, Vienna, Austria 2015.

[73] Harrison PM. Exhaustive assignment of compositional bias reveals universally prevalent biased regions: analysis of functional associations in human and Drosophila. BMC bioinformatics. 2006;7:441 10.1186/1471-2105-7-441 ; PubMed Central PMCID: PMC1618407. 17032452 PMC1618407

[74] Caron C, Boyault C, Khochbin S. Regulatory cross-talk between lysine acetylation and ubiquitination: role in the control of protein stability. Bioessays. 2005;27(4):408–15. 10.1002/bies.20210 WOS:000228041600008. 15770681

[75] Iakoucheva LM, Radivojac P, Brown CJ, O'Connor TR, Sikes JG, Obradovic Z, et al The importance of intrinsic disorder for protein phosphorylation. Nucleic Acids Res. 2004;32(3):1037–49. 10.1093/nar/gkh253 ; PubMed Central PMCID: PMCPMC373391. 14960716 PMC373391

[76] Wu Z, Connolly J, Biggar KK. Beyond histones—the expanding roles of protein lysine methylation. FEBS J. 2017 Epub 2017/03/16. 10.1111/febs.14056 . 28294537

[77] Fukuchi S, Sakamoto S, Nobe Y, Murakami SD, Amemiya T, Hosoda K, et al IDEAL: Intrinsically Disordered proteins with Extensive Annotations and Literature. Nucleic acids research. 2012;40(Database issue):D507–11. 10.1093/nar/gkr884 ; PubMed Central PMCID: PMC3245138. 22067451 PMC3245138

[78] Mohan A, Oldfield CJ, Radivojac P, Vacic V, Cortese MS, Dunker AK, et al Analysis of molecular recognition features (MoRFs). J Mol Biol. 2006;362(5):1043–59. Epub 2006/08/29. 10.1016/j.jmb.2006.07.087 . 16935303

[79] Dyson HJ, Wright PE. Coupling of folding and binding for unstructured proteins. Current opinion in structural biology. 2002;12(1):54–60. . 11839490 10.1016/s0959-440x(02)00289-0

[80] Hasan S, Stucki M, Hassa PO, Imhof R, Gehrig P, Hunziker P, et al Regulation of human flap endonuclease-1 activity by acetylation through the transcriptional coactivator p300. Molecular Cell. 2001;7(6):1221–31. 10.1016/S1097-2765(01)00272-6 WOS:000169547400010. 11430825

[81] Trexler AJ, Rhoades E. N-terminal acetylation is critical for forming a-helical oligomer of a-synuclein. Protein Science. 2012;21(5):601–5. 10.1002/pro.2056 WOS:000302620600001. 22407793 PMC3403458

[82] Huntley M, Golding GB. Evolution of simple sequence in proteins. Journal of molecular evolution. 2000;51(2):131–40. WOS:000088831500004. 10948269 10.1007/s002390010073

[83] Faux NG, Bottomley SP, Lesk AM, Irving JA, Morrison JR, de la Banda MC, et al Functional insights from the distribution and role of homopeptide repeat-containing proteins. Genome Res. 2005;15(4):537–51. 10.1101/gr.3096505 WOS:000228203000010. 15805494 PMC1074368

[84] Alba MM, Tompa P, Veitia RA. Amino acid repeats and the structure and evolution of proteins. Genome Dyn. 2007;3:119–30. Epub 2008/08/30. 10.1159/000107607 . 18753788

[85] Otvos L Jr., Cudic M. Post-translational modifications in prion proteins. Curr Protein Pept Sci. 2002;3(6):643–52. Epub 2002/12/10. . 12470218 10.2174/1389203023380440

[86] Gendoo DMA, Harrison PM. The Landscape of the Prion Protein's Structural Response to Mutation Revealed by Principal Component Analysis of Multiple NMR Ensembles. PLoS computational biology. 2012;8(8). ARTN e1002646 10.1371/journal.pcbi.1002646 WOS:000308553500031. PMC3415401 22912570

[87] Harrison PM, Khachane A, Kumar M. Genomic assessment of the evolution of the prion protein gene family in vertebrates. Genomics. 2010;95(5):268–77. 10.1016/j.ygeno.2010.02.008 WOS:000277258700005. 20206252

[88] Waterhouse AM, Procter JB, Martin DM, Clamp M, Barton GJ. Jalview Version 2—a multiple sequence alignment editor and analysis workbench. Bioinformatics. 2009;25(9):1189–91. Epub 2009/01/20. 10.1093/bioinformatics/btp033 ; PubMed Central PMCID: PMCPMC2672624. 19151095 PMC2672624

[89] Dai TY, Cao L, Yang ZC, Li YS, Tan L, Ran XZ, et al P68 RNA helicase as a molecular target for cancer therapy. J Exp Clin Canc Res. 2014;33 ARTN 64 10.1186/s13046-014-0064-y WOS:000344696400001. PMC4431487 25150365

[90] Kontaxi C, Piccardo P, Gill AC. Lysine-Directed Post-translational Modifications of Tau Protein in Alzheimer's Disease and Related Tauopathies. Front Mol Biosci. 2017;4:56 Epub 2017/08/30. 10.3389/fmolb.2017.00056 ; PubMed Central PMCID: PMCPMC5554484. 28848737 PMC5554484

[91] Rathert P, Dhayalan A, Murakami M, Zhang X, Tamas R, Jurkowska R, et al Protein lysine methyltransferase G9a acts on non-histone targets. Nat Chem Biol. 2008;4(6):344–6. Epub 2008/04/29. 10.1038/nchembio.88 ; PubMed Central PMCID: PMCPMC2696268. 18438403 PMC2696268

[92] Wesche J, Kuhn S, Kessler BM, Salton M, Wolf A. Protein arginine methylation: a prominent modification and its demethylation. Cell Mol Life Sci. 2017 Epub 2017/04/02. 10.1007/s00018-017-2515-z . 28364192 PMC11107486

[93] Ong SE, Mittler G, Mann M. Identifying and quantifying in vivo methylation sites by heavy methyl SILAC. Nat Methods. 2004;1(2):119–26. Epub 2005/03/23. 10.1038/nmeth715 . 15782174

[94] Banerjee K, Wang M, Cai E, Fujiwara N, Baker H, Cave JW. Regulation of tyrosine hydroxylase transcription by hnRNP K and DNA secondary structure. Nat Commun. 2014;5 ARTN 5769 10.1038/ncomms6769 WOS:000347612800003. 25493445 PMC4264680

[95] Choudhary C, Kumar C, Gnad F, Nielsen ML, Rehman M, Walther TC, et al Lysine Acetylation Targets Protein Complexes and Co-Regulates Major Cellular Functions. Science. 2009;325(5942):834–40. 10.1126/science.1175371 WOS:000269242400036. 19608861

[96] Mooney SM, Grande JP, Salisbury JL, Janknecht R. Sumoylation of p68 and p72 RNA Helicases Affects Protein Stability and Transactivation Potential. Biochemistry. 2010;49(1):1–10. 10.1021/bi901263m WOS:000273267300001. 19995069

[97] Gilberto S, Peter M. Dynamic ubiquitin signaling in cell cycle regulation. J Cell Biol. 2017;216(8):2259–71. 10.1083/jcb.201703170 WOS:000407078100009. 28684425 PMC5551716

[98] Bannister AJ, Kouzarides T. Regulation of chromatin by histone modifications. Cell Res. 2011;21(3):381–95. Epub 2011/02/16. 10.1038/cr.2011.22 ; PubMed Central PMCID: PMCPMC3193420. 21321607 PMC3193420

[99] Beltrao P, Bork P, Krogan NJ, van Noort V. Evolution and functional cross-talk of protein post-translational modifications. Mol Syst Biol. 2013;9:714 Epub 2013/12/25. 10.1002/msb.201304521 ; PubMed Central PMCID: PMCPMC4019982. 24366814 PMC4019982

[100] Wisniewski JR, Zougman A, Kruger S, Mann M. Mass spectrometric mapping of linker histone H1 variants reveals multiple acetylations, methylations, and phosphorylation as well as differences between cell culture and tissue. Molecular & Cellular Proteomics. 2007;6(1):72–87. 10.1074/mcp.M600255-MCP200 WOS:000243312000007. 17043054

[101] Hergeth SP, Schneider R. The H1 linker histones: multifunctional proteins beyond the nucleosomal core particle. Embo Rep. 2015;16(11):1439–53. WOS:000364318900010. 10.15252/embr.201540749 26474902 PMC4641498

[102] Parkel S, Lopez-Atalaya JP, Barco A. Histone H3 lysine methylation in cognition and intellectual disability disorders. Learn Memory. 2013;20(10):570–9. 10.1101/lm.029363.112 WOS:000325860600007. 24045506

[103] Cao J, Yan Q. Histone ubiquitination and deubiquitination in transcription, DNA damage response, and cancer. Front Oncol. 2012;2:26 10.3389/fonc.2012.00026 ; PubMed Central PMCID: PMCPMC3355875. 22649782 PMC3355875

[104] Sears R, Nuckolls F, Haura E, Taya Y, Tamai K, Nevins JR. Multiple Ras-dependent phosphorylation pathways regulate Myc protein stability. Gene Dev. 2000;14(19):2501–14. 10.1101/gad.836800 WOS:000089765400009. 11018017 PMC316970

[105] Qin WH, Wolf P, Liu N, Link S, Smets M, La Mastra F, et al DNA methylation requires a DNMT1 ubiquitin interacting motif (UIM) and histone ubiquitination. Cell Research. 2015;25(8):911–29. 10.1038/cr.2015.72 WOS:000358941100006. 26065575 PMC4528052

[106] Carbon S, Dietze H, Lewis SE, Mungall CJ, Munoz-Torres MC, Basu S, et al Expansion of the Gene Ontology knowledgebase and resources. Nucleic Acids Research. 2017;45(D1):D331–D8. 10.1093/nar/gkw1108 WOS:000396575500049. 27899567 PMC5210579

