# Supplementary material for: Discerning evolutionary trends in post-translational modification and the effect of intrinsic disorder: Analysis of methylation, acetylation and ubiquitination sites in human proteins
Source: PLoS Comput Biol. 2018 Aug 10;14(8):e1006349. doi: 10.1371/journal.pcbi.1006349 (PMC6105011; doi:10.1371/journal.pcbi.1006349)
Supplement: S2 File — The numbers in the bubbles are the fraction of conserved MAU sites with the number of conserved sites and P-value showing the enrichment of conserved sites in ordered and disordered regions at each level and the fraction of new conserved MAU sites with the number of new conserved sites in ordered and disordered regions at each level. (A) Fraction of conserved and new conserved lysine methylation sites and the number of conserved and new conserved lysine methylation sites in ordered and disordered regions. (B) Fraction of conserved and new conserved arginine methylation sites and the number of conserved and new arginine methylation sites in ordered and disordered regions. (C) Fraction of conserved and new conserved lysine acetylation sites and the number of conserved and new lysine acetylation sites in ordered and disordered regions. (D) Fraction of conserved and new conserved lysine ubiquitination sites and the number of conserved and new lysine ubiquitination sites in ordered and disordered regions. (DOCX) [file pcbi.1006349.s002.docx]

**Supplementary S2 File: Evolutionary trees showing the conservation of MAU-site residues in ordered and disordered regions at each eukaryotic clade.**

The bubble size in the evolutionary trees represents the fraction of conserved and newly-emerged MAU sites in ordered (olive green) and disordered (peach) regions at each clade. A hypergeometric probability test is applied to identify the enrichment of conserved and new conserved MAU sites in ordered and disordered regions at each clade. The test is performed with all lysine/arginine residues in the proteins with MAU sites set as background population, all conserved lysine/arginine residues as success in background population, MAU-modified lysine/arginine residues as sample and conserved MAU-modified sites as success in sample. We applied a Bonferroni correction for multiple hypothesis testing and the P-values are considered significant at P < 0.00417 for lysine modifications and P < 0.0125 arginine methylation. The number of conserved MAU sites at each clade and the significance of enriched conserved and newly-emerged MAU sites in ordered and disordered regions are given inside the bubbles.

Figure A: Conserved lysine methylation sites and new conserved sites in ordered and disordered regions at each eukaryotic level

Figure B: Conserved arginine methylation sites and new conserved sites in ordered and disordered regions at each eukaryotic level

Figure C. Conserved lysine acetylation sites and new conserved sites in ordered and disordered regions at each eukaryotic level

Figure D. Conserved ubiquitination sites and new conserved sites in ordered and disordered regions at each eukaryotic level
