# Supplementary material for: Discerning evolutionary trends in post-translational modification and the effect of intrinsic disorder: Analysis of methylation, acetylation and ubiquitination sites in human proteins
Source: PLoS Comput Biol. 2018 Aug 10;14(8):e1006349. doi: 10.1371/journal.pcbi.1006349 (PMC6105011; doi:10.1371/journal.pcbi.1006349)
Supplement: S3 File — Summary of Gene Ontology (GO) category enrichments at different evolutionary levels. (A) Gene ontology category enrichments for proteins with newly emerged conserved MAU-site residues at each evolutionary level are depicted in heat map form. (B) Gene Ontology category for the whole set of MAU-modified proteins. (DOCX) [file pcbi.1006349.s003.docx]

**S3 File: Gene Ontology enrichments**

Gene ontology (GO) category enrichments for proteins with newly emerged conserved MAU-site residues at each evolutionary level are depicted in heat map form in Figure part A below. The heat map colour indicates the value of the log P-value for significant GO category enrichments.

In Figure part B, the same calculation is performed for the whole set of MAU-modified proteins.

These calculations were performed as described in the *Methods* section.

A

B
